# Design, Synthesis, and Cellular Characterization of a New Class of IPMK Kinase Inhibitors

**DOI:** 10.1021/acs.jmedchem.5c00015

**Published:** 2025-07-25

**Authors:** Yubai Zhou, Pratima Chapagain, Desmarini Desmarini, Dilipkumar Uredi, Michael A. Stashko, Hundaol Huluka, Lucia E. Rameh, Julianne T. Djordjevic, Raymond D. Blind, Xiaodong Wang

**Affiliations:** † Center for Integrative Chemical Biology and Drug Discovery, Division of Chemical Biology and Medicinal Chemistry, Eshelman School of Pharmacy, 2331 University of North Carolina at Chapel Hill, Chapel Hill, North Carolina 27599, United States; ‡ Department of Medicine, Division of Diabetes, Endocrinology & Metabolism, 5557 Vanderbilt University Medical Center, Nashville, Tennessee 37232, United States; § Departments of Biochemistry & Pharmacology, Vanderbilt University School of Medicine, Nashville Tennessee 37232, United States; ∥ Centre for Infectious Diseases and Microbiology, 107640 The Westmead Institute for Medical Research, Westmead NSW 2145, Australia; ⊥ Sydney Institute for Infectious Diseases, Faculty of Medicine and Health, University of Sydney, Sydney NSW 2006, Australia; # Department of Biochemistry & Molecular Biology, University of South Alabama, Mobile, Alabama 36688, United States; ○ Lineberger Comprehensive Cancer Center, University of North Carolina at Chapel Hill, Chapel Hill, North Carolina 27599, United States

## Abstract

The kinase activity of human inositol phosphate multikinase (IPMK) is required for the synthesis of higher-order inositol phosphate signaling molecules, regulation of gene expression, and control of the cell cycle. Here, we report a novel series of highly potent IPMK inhibitors. The first-generation IPMK inhibitor **1** (UNC7437) decreased cellular proliferation and tritiated inositol phosphate levels in metabolically labeled human U251-MG glioblastoma cells. It also impacted the transcriptome of these cells, selectively regulating 993 genes enriched in cancer, epithelial-to-mesenchymal transition (EMT), and inflammatory and viral infection pathways, consistent with anticancer growth activity. Extensive optimization of **1** led to **14** (UNC9750) with improved pharmacokinetic properties. Compound **14** inhibited cellular accumulation of InsP_5_, the direct product of IPMK kinase activity, while having no effect on either InsP_6_ or InsP_7_ levels. These studies suggest that rapid chemical inhibition of IPMK induces a novel InsP_5_ metabolic signature, providing new biological insights into inositol phosphate metabolism and signaling.

## Graphical abstract

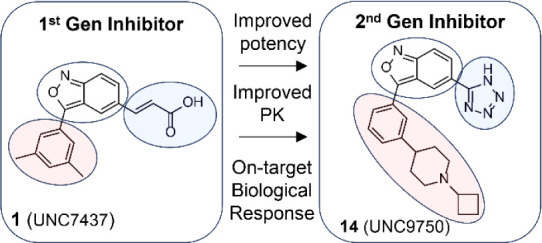

## Introduction

The human inositol phosphate multikinase (IPMK) is a promiscuous kinase that can phosphorylate several species of InsP_4_ and InsP_5_ that are required for full InsP_6_ production^1–4^ and the membrane phospholipid PI­(4,5)­P_2_ ^5,6^ to generate PIP_3_ in membranes,^2,5^ which are all biologically important metabolites and/or small signaling molecules. IPMK can also directly phosphorylate transcription factor-bound PI­(4,5)­P_2_.^6^ As might be expected with such a diverse set of kinase activities, the genetic loss of IPMK results in significant changes to many important cellular processes, including but not limited to cell proliferation and gene expression. Genetic studies suggest that the kinase activity of IPMK controls many aspects of these complex processes. However, the role of the kinase activity in these processes, as well as the investigation of the kinetics and functional consequences of IPMK metabolites in cells, must be validated by developing specific inhibitors of IPMK.

Genetic loss of IPMK is embryonic lethal in flies and mice,^7,8^ suggesting that the clinical use of IPMK inhibitors might carry detrimental side effects. However, several lines of evidence suggest that IPMK inhibitors will be well tolerated in humans. Flies and mice heterozygous for IPMK loss bear no detectable phenotypes^7,8^ and mutant hypomorphic IPMK alleles from plants can rescue developmental loss of IPMK in animals.^7,9^ Orthologous IPMK alleles from various species, including the plant , can rescue loss of IPMK function in animal development.^7^ These data suggest that only a small fraction of the total IPMK kinase activity is required for proper embryonic development in animals. Tissue-specific IPMK knockout mice in the liver, adipocytes, and brain are viable, and while these mice have revealed important physiological insights,^10–12^ the adults show no increased mortality. The developmental defect in whole-animal IPMK-knockout mice occurs early in development (E9.5), specifically during neural tube closure.^8^ Thus, the genetic data suggest that a small fraction of IPMK kinase activity is essential during vertebrate development and that IPMK loss should be well tolerated in adult animals. Of course, this conclusion awaits chemical validation using IPMK inhibitors in preclinical adult animal models.

IPMK has also been linked to cancer in several studies.^5,13–20^ Loss of IPMK kinase activity in cells is associated with decreased proliferation. Thus, it has been speculated that IPMK inhibitors may have utility as anticancer therapies. There is also evidence suggesting that inhibiting IPMK could be therapeutically beneficial in some cancers that are phosphatase and tensin homologue (PTEN)-negative and grow in an AKT-independent manner.^6^ IPMK localizes to the nucleus, where it can activate gene expression in a kinase-dependent manner.^5,6,21–23^ IPMK-activated gene expression can be antagonized by the tumor suppressor PTEN in a phosphatase-dependent manner, with IPMK functioning as the “on” switch and PTEN as the opposing “off” switch in these transcriptional studies.^6^ Transcriptional activation by IPMK was not recapitulated by the classic PI3K p110α, and the PI3K inhibitor wortmannin did not recapitulate the effects of attenuating IPMK activity, suggesting that PTEN is not antagonizing p110 PI3-kinase activity, but rather the activity of IPMK.^6^ This phosphatase-dependent nuclear PTEN activity has clinical implications—if the growth of PTEN-negative tumors is driven by nuclear PTEN function in transcriptional regulation,^24^ these tumors might respond to an IPMK inhibitor, but not to inhibitors of the p110 PI3-kinases. Indeed, almost 20 years ago, decreased proliferation of the PTEN-negative human glioblastoma cell line U251-MG was observed only when wild-type PTEN was expressed in the nucleus, while cytoplasmic PTEN had no effect on U251-MG cell growth.^24^ Further, nuclear PTEN expression had no effect on AKT phosphorylation, suggesting the nuclear PTEN pathway is decoupled from PI3-kinase signaling,^24^ despite several lines of evidence suggesting that IPMK regulates AKT in other cellular systems.^5^ Taken together, although PTEN loss can drive cancer through several mechanisms, these data support the development of inhibitors against IPMK, which could be effective in halting the growth of some PTEN-negative tumors modeled by U251-MG cancer cells, particularly human glioblastomas.

Here, we report the development of first- and second-generation IPMK inhibitors. We also characterize their effects on human U251-MG glioblastoma cells and their pharmacokinetic properties in mice. Compound **14** had the best pharmacokinetic properties, and treatment of human U251-MG glioblastoma cells with this compound produced a novel metabolic profile not previously observed in genetic loss-of-function studies of IPMK: Compound **14** decreased InsP_5_ levels, without affecting InsP_6_ abundance in ^3^H-inositol-labeled U251 glioblastoma cells. Our data suggest that chemical inhibition of IPMK can reveal novel metabolic profiles in human glioblastoma cells that were not previously observed using genetic approaches. The compounds presented here will form the basis of future efforts to develop IPMK inhibitors with the potential for clinical applications, particularly in human glioblastoma.

## Results

### Compound **1** (UNC7437) Inhibits IP Levels and Human Glioblastoma Cell Line Proliferation

IPMK is a key enzyme in higher-order inositol phosphate biosynthesis (Figure 1A). In our efforts to develop IP6K inhibitors,^25^ we fortuitously discovered that compound **1** had off-target efficacy toward IPMK kinase activity. Here, we report the biological characterization of compound **1** (Figure 1B), with an IC_50_ of 26.2 nM toward pure, recombinant human IPMK, as determined by using a Kinase-Glo assay. Several previous studies have used genetic analyses to link the kinase activity of IPMK with gene expression and transcriptional regulation.^6^ We therefore asked if rapid treatment with compound **1** might regulate gene expression in a cell-based model of human cancer, the human glioblastoma cell line U251-MG (U251). Treatment with 400 nM compound **1** (15× greater than the IC_50_) resulted in a significant decrease in cell number, as determined using a CyQuant DNA content assay (Figure 1C). Identical treatment of these cells with compound **1** significantly decreased the accumulation of InsP_4_, InsP_5_ and InsP_6_, as measured by ^3^H-inositol labeling followed by HPLC (Figure 1D,E). Taken together, these data suggest that compound **1** decreased the proliferation and survival of U251 glioblastoma cells and inhibited inositol phosphate synthesis.

**Figure 1:**
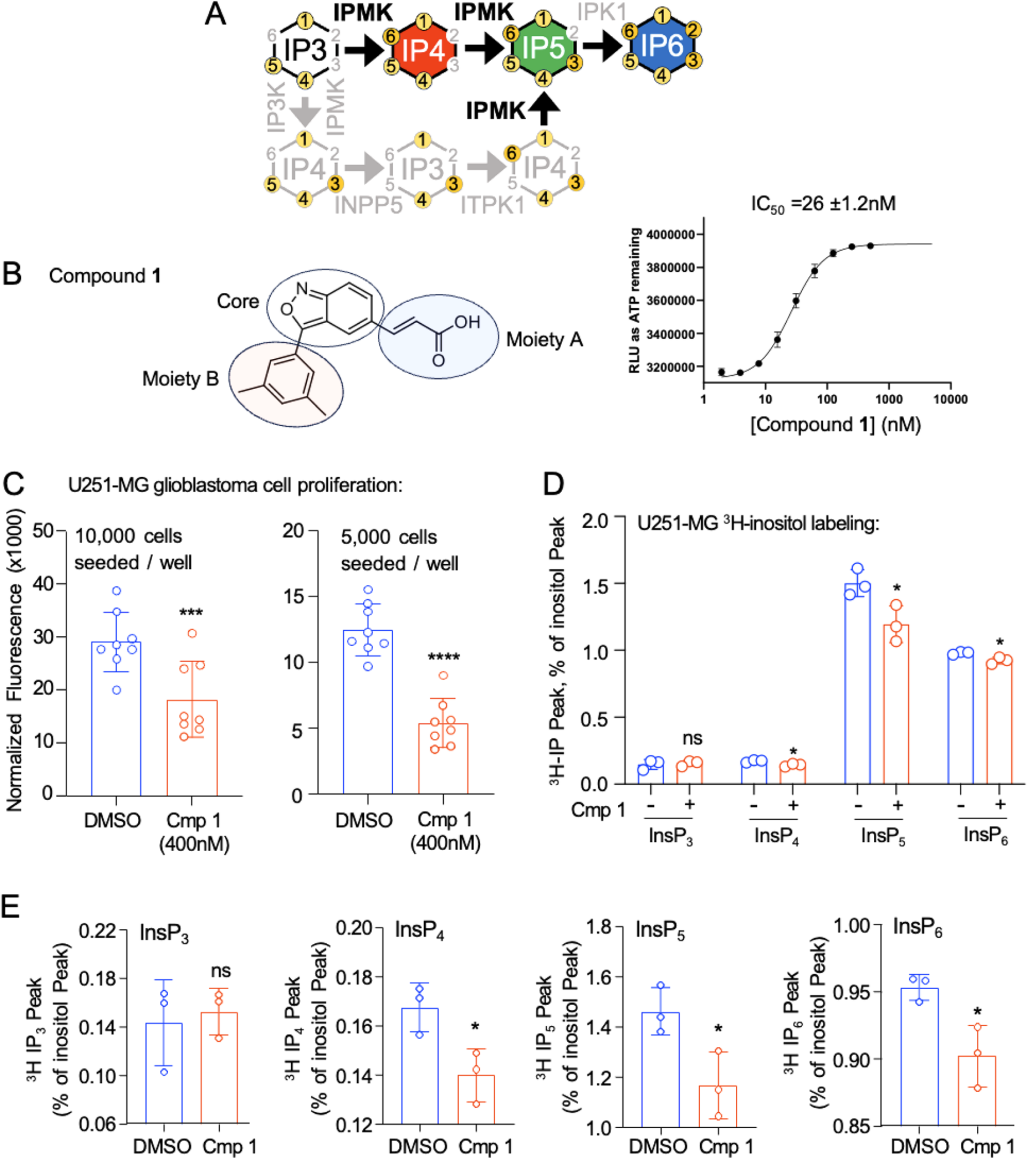
Compound **1** inhibits human U251 glioblastoma cell growth and labeled inositol phosphate levels. (A) Schematic of inositol phosphate metabolism. (B) Chemical structure of compound **1**, indicating moiety A (blue highlight), the core (gray highlight), and moiety B (red highlight), and the IC50 value as determined by the kinase-GLO assay. (C) CyQuant cell count assay of 10,000 human U251-MG glioblastoma cells seeded per well, treated for 48 h with DMSO vehicle or 400 nM compound **1** (*n* = 8, ***p = 0.0046, unpaired *t* test). (D) HPLC analysis of ^3^H-inositol phosphates from U251-MG cells treated with 400 nM compound **1** (red bars) or DMSO (blue bars) and metabolically labeled with ^3^H-inositol for 48 h, expressed as the percentage of the inositol peak from the HPLC for InsP3, InsP4, InsP5, and InsP6, as indicated, *p < 0.05 by unpaired *t* test Bonferroni corrected for multiple comparisons. (E) Same data as in (D) replotted with a nonzero *y*-axis to facilitate visualizing differences between DMSO vs. compound treatment for each indicated inositol phosphate species, *p < 0.05 by unpaired *t* test Bonferroni corrected for multiple comparisons.

### Compound **1** Regulates Genes Important for Cell Growth and Inflammatory Responses

IPMK and inositol phosphates have been linked to transcriptional regulation,^21,26–30^ so we performed RNA-seq transcriptome analysis on cells treated for 48 h with 400 nM compound **1** vs. DMSO control (Figure 2A). Transcriptome analyses identified 993 differentially expressed transcripts (*p*_adj_ < 0.05) with a log 2 fold change (log_2_FC) > ±1. Among these genes, 661 transcripts were downregulated, while 332 were upregulated. We subjected the differentially expressed transcripts to gene set enrichment analysis (GSEA) using the Hallmark Pathways collection from the Molecular Signatures Database (Figure 2B), revealing several pathways associated with cancer or tumor metastasis that were significantly enriched with the gene sets regulated by compound **1** (apical junction, mitotic spindle, epithelial–mesenchymal transition (EMT), MYC targets, and KRAS signaling). A similar GSEA analysis using all gene sets in the Molecular Signatures Database identified enrichment of genes belonging to two categories: (1) cancer gene sets and (2) viral/inflammation gene sets (Figure S1), although other gene sets were also significantly enriched as well; see the Supporting Information for the full list of all enriched gene sets. Compound **1**-regulated transcripts were enriched in the Verhaak-Glioblastoma Classical gene set (Figure 2C) and in NFKBIA target genes (Figure S1), while compound **1**-regulated transcripts were significantly de-enriched in several virus, vaccine, and apoptosis gene sets (Figure 2D, and Figure S1). Together, these data suggest that compound **1** decreases the proliferation of these human U251 glioblastoma cancer cells and alters the expression of genes associated with cancer and inflammatory pathways.

**Figure 2:**
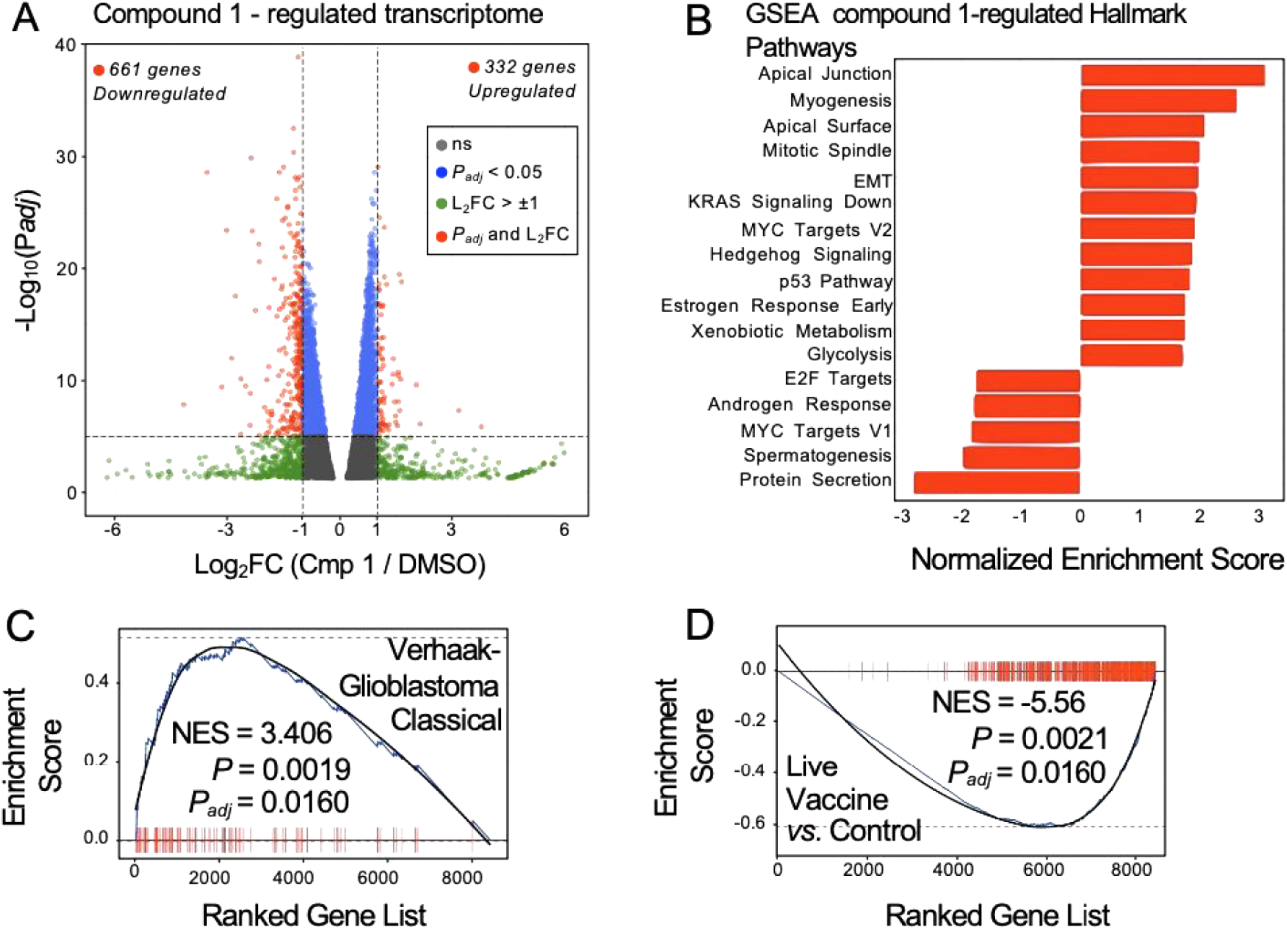
Compound **1** alters genes in glioblastoma cells enriched in cancer, EMT, and inflammatory pathways. (A) Volcano plot from biological triplicate RNA-seq of human U251-MG glioblastoma cells, showing differentially expressed transcripts upon treatment with 400 nM compound **1** for 48 h, compared to the DMSO vehicle control. (B) Gene set enrichment analysis (GSEA) of the compound **1**-differentially expressed gene set showed enrichment in several hallmark cancer pathways. (C) Bar code GSEA plots from representative enriched and (D) de-enriched gene sets using the Molecular Signatures Database.

### Modification of Moiety A to Improve Potency and Drug-like Properties

We then sought to increase the potency, efficacy, and drug-like properties of compound **1** through medicinal chemistry. We noted the double bond on the acrylic acid side chain in **1** may be a potential Michael acceptor, which could bind proteins nonselectively through a cysteine residue and lead to toxicity.^31^ Therefore, this double bond was removed either by introducing saturation or being truncated from the molecule. We first replaced the double bond with a saturated cyclopropyl ring to yield compound **2** (Figure 3A,B). As depicted in Scheme 1A, a Corey–Chaykovsky cyclopropanation of the known compound **5** with a sulfonium ylide formed *in situ* from trimethylsulfoxonium iodide, followed by an ester hydrolysis reaction under basic conditions, yielded the desired compound **2**. Compound **2** had a 6-fold decreased inhibitory activity toward IPMK. It also contains two stereocenters that would likely necessitate separation into pure enantiomers (Figure 3A). On the other hand, when we truncated the acrylic acid side chain to a carboxylic acid group, the resulting compound **3** (Figure 3B) retained inhibitory activity toward IPMK with a much shorter synthesis (1 step vs. 4-step for **1**, see Supporting Information)^25^ although it was 8-fold weaker compared to **1**.^25^ Therefore, we decided to resolve the liability of being a potential Michael acceptor by the truncation of the acrylic acid side chain to a carboxylic acid group. Furthermore, the carboxylic acid functional group often leads to lower permeability to biological membranes and is thus frequently replaced by an acid bioisostere.^32,33^ In our effort to identify a suitable acid bioisostere to replace the carboxylic acid functional group in compound **3**, we found that compound **4**, containing a tetrazole, displayed improved potency (9-fold) and solubility compared to compound **3** (Figure 3C). Therefore, tetrazole was chosen as an optimized group at moiety A and will be retained for further optimization at moiety B. Also worth noting is that the synthesis of compound **4** is straightforward since both starting materials, 2-(3,5-dimethylphenyl)­acetonitrile and 5-(4-nitrophenyl)-1*H*-tetrazole, are commercially available (Scheme 1). A ring formation reaction between 2-(3,5-dimethylphenyl)­acetonitrile and 5-(4-nitrophenyl)-1*H*-tetrazole under basic conditions yielded the desired compound **4** with a 26% yield. This cyclization reaction is also the key step for other tetrazole analogue syntheses and can be performed on a multigram scale.

**Figure 3:**
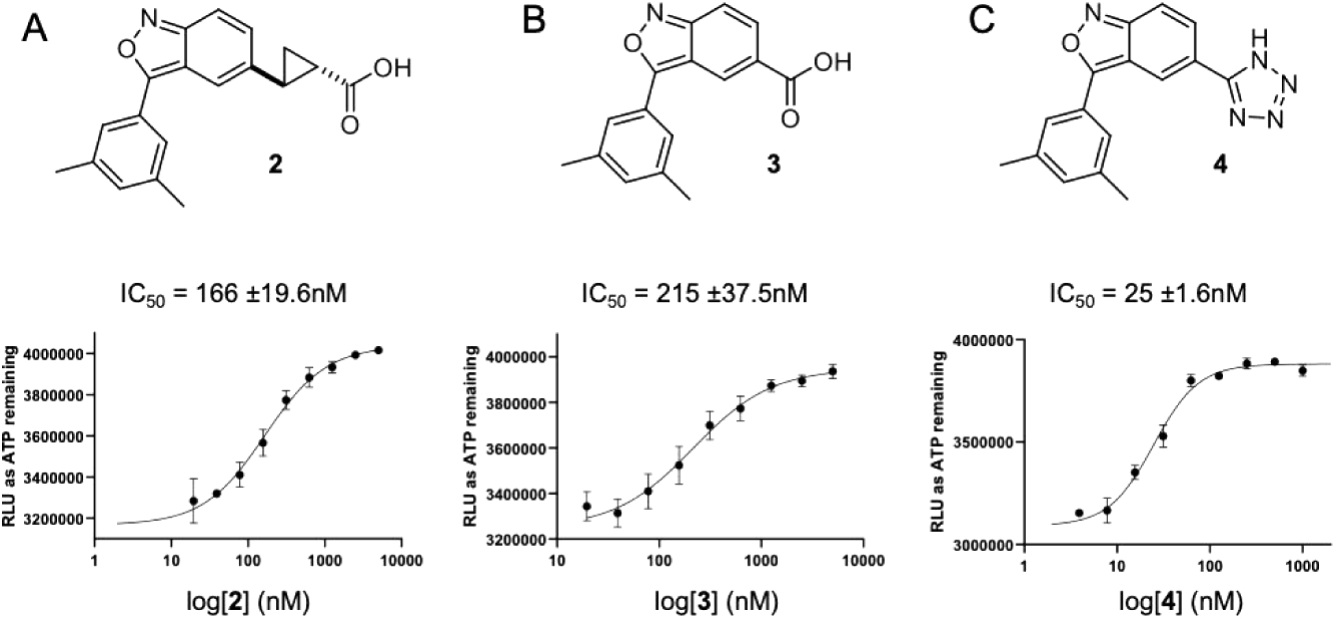
Compounds **2**, **3**, and **4** inhibit the kinase activity of human IPMK. Chemical structures, IC_50_ values, and curve fits for (A) compound **2**, (B) compound **3**, and (C) compound **4**.

**Scheme 1:**
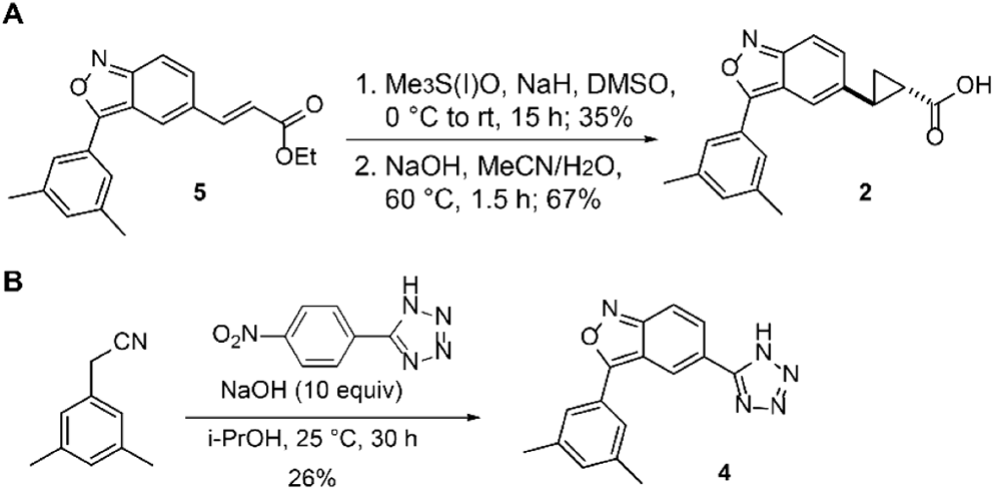
Synthetic Scheme for Compound **2** and Compound **4**.; Synthesis of (A) Compound **2** and (B) Compound **4**

### Modification of Moiety B to Improve Potency and Drug-like Properties

Next, we explored the structure–activity relationships at the R position (moiety B). A variety of groups were introduced at this position, as illustrated in Figure 4. Removal of the 3,5-dimethyl groups on the phenyl ring yielded a slightly weaker compound **6** (Figure 4B) compared to compound **4**. Both 4-trifluoromethylphenyl and 4-trifluoromethoxyphenyl groups at the R position slightly increased the potency of the corresponding compounds **7** (Figure 4C) and **8** (Figure 4D). However, a larger 4-phenoxyphenyl group at this site significantly reduced the activity to yield compound **9** (Figure 4E). A 4-(cyclopropanecarboxamido)­phenyl group led to a weaker compound **10** (Figure 4F), while a 3-(cyclopropanecarboxamido)­phenyl group yielded a similarly potent compound **11** (Figure 4G). Overall, most of the above-specified chemical groups were well tolerated at moiety B, which gives us an opportunity to modify the pharmacokinetic (PK) properties of these compounds. Therefore, two potent and structurally different compounds **8** and **11** were evaluated in mice for PK properties via intravenous (IV) administration at a 3 mg/kg dose (Table 1).

**Figure 4:**
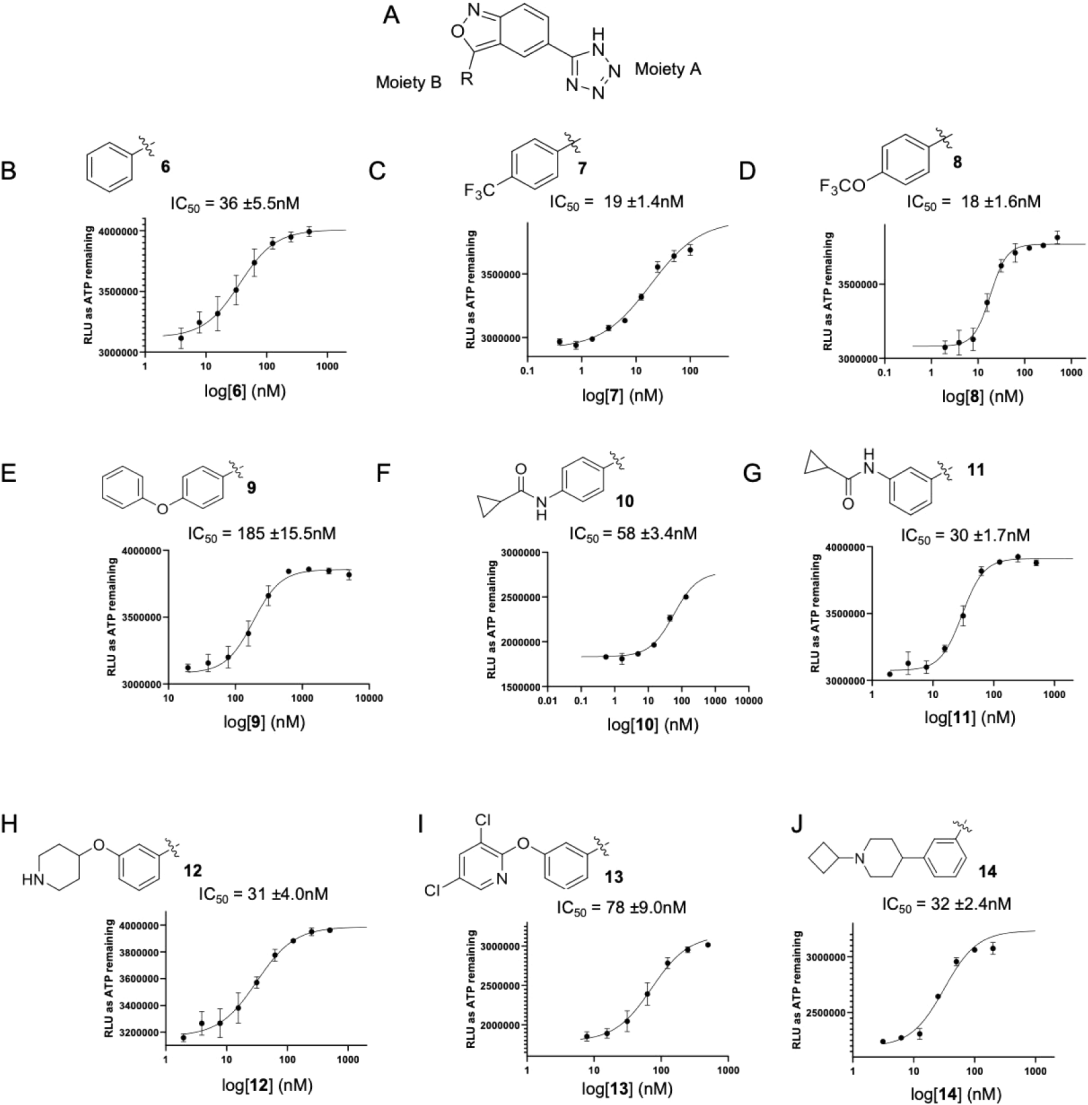
Compounds **6**–**12** inhibit the kinase activity of human IPMK. Chemical structure of the (A) backbone scaffold of compound **4** with moiety B as the R group and moiety A and the core indicated. Chemical structures of the R groups (moiety B) are compounds **6** (B), **7** (C), **8** (D), **9** (E), **10** (F), **11** (G), **12** (H), **13** (I), and **14** (J).

**Table 1:** *In Vivo* Pharmacokinetic Parameters of Selected Analogues (3 mg/kg, *N* = 2 Mice per Time Point)

Compound^a^	*T*_1/2_ (h)	Cmax (μM)	AUClast (h·μM)	Vss (L/kg)	CL (mL/min/kg)
**8**	0.92	33.0	46.9	0.26	3.15
**11**	1.09	66.6	19.4	0.21	7.52
**12**	1.29	44.0	10.8	0.23	11.8
**13**	2.01	50.5	7.7	0.27	15.5
**14** ^b^	1.95	28.3	4.6	0.39	22.8

a Intravenous formulation: 10% NMP, 5% solution in normal saline (0.9% NaCl).

b Dose: 2.75 mg/kg.

Both compounds had a low clearance (CL) (<7.5 mL/min/kg); however, they exhibited a short half-life of around 1 h due to a low volume of distribution (Vss, <0.26 L/kg, less than the mouse whole-body water content of 0.70 L/kg).^34^ To increase the volume of distribution, we next attempted to introduce basic groups into these inhibitors since basic groups intend to increase the volume of distribution.^34^ Compounds **12–14** incorporated basic groups such as 4-piperidine (**12**, Figure 4H), 3,5-dichloropyridine (**13**, Figure 4I) and 4-(1-cyclobutyl)­piperidinyl (**14**, Figure 4J) at the *meta*-position of the phenyl ring, and all compounds retained potent inhibitory activity toward IPMK. The volume of distribution was increased for compounds **13** and **14** (Table 1). Compound **14** had a larger volume of distribution (0.39 L/kg) and a longer half-life (1.95 h) while retaining low clearance (22.8 mL/min/kg); thus, it was further evaluated in mice with a 10 mg/kg dose via IV and intraperitoneal (IP) administration (*n* = 3). Compound **14** had a large AUClast (8.26 h·μM) and 82% IP bioavailability (Figure 5). We also evaluated specificity for IPMK over IP6K2, with all compounds **1**–**14** being selective for IPMK to varying degrees, except compound **3**, which was selective for IP6K2 (Table S1). Compound **14** was the most specific for IPMK, with 12-fold selectivity for IPMK over IP6K2 (Table S1), and since compound **14** displays good *in vivo* PK properties suggesting potential *in vivo* utility, **14** was further evaluated in cell-based assays.

**Figure 5:**
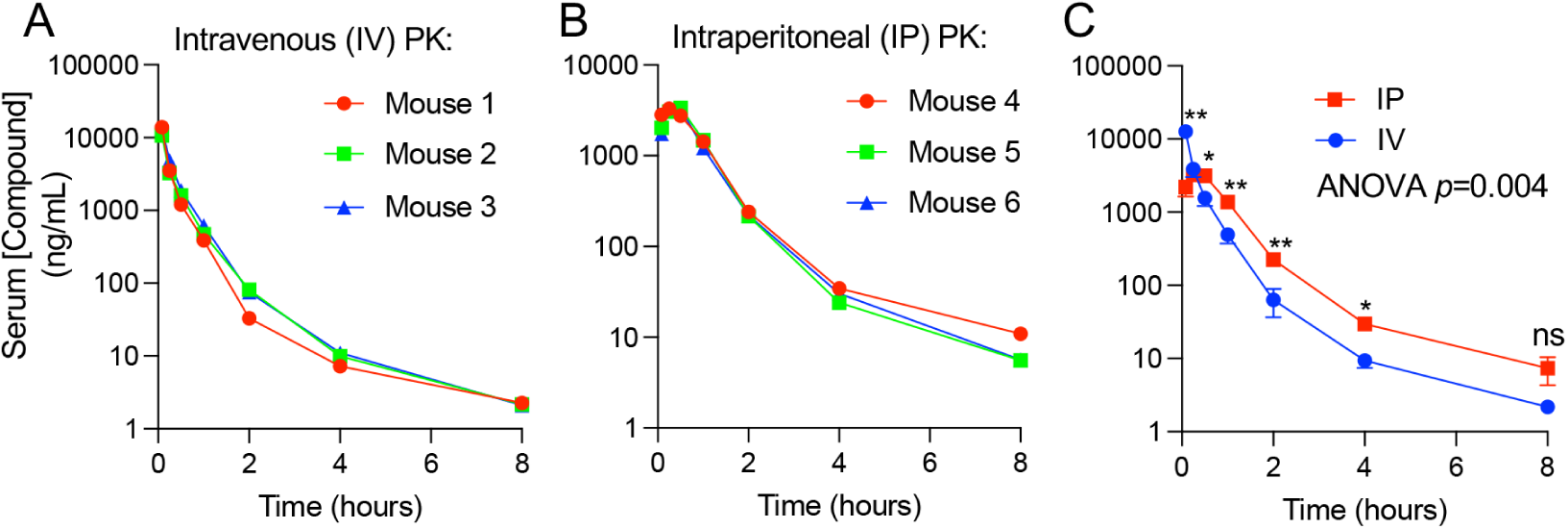
Pharmacokinetic study of compound **14**. Serum concentration of the indicated compound in ng/mL of (A) intravenous (IV) or (B) intraperitoneal (IP) injected **14** measured in three independent animals. (C) Overlay of PK curves from mean IP (red) vs. mean IV (blue) administration, error shown is the SD of the mean, no significant (ns) differences between IP vs. IV time points were detected at 0.25 h and 8 h, and differences were detected at all other times as indicated (*p ≤ 0.05, **p ≤ 0.01 by Holm–Sidak corrected *t* tests), and across all time points in IP vs. IV in two-factor ANOVA (*p* = 0.004).

### Compound **14** Decreases Human Glioblastoma Cell Growth and Selectively Reduces Cellular InsP_5_ Abundance

We next tested the effect of compound **14** (Figure 6A) on various functional aspects of the human glioblastoma cancer cell line U251-MG. Compound **14** decreased cell growth (1.0 μM, 48 h), with a more dramatic effect occurring at a higher dose. Using ^3^H-inositol metabolically labeled cells treated with 10 μM compound **14** for 48 h, InsP_4_ levels increased by about 50% which was statistically significant, while InsP_5_ levels decreased by 50% which was more robustly significant. Importantly, we observed no change in the level of InsP_6_ (Figure 6B–D). The unexpected lack of change in InsP_6_ contrasts with results obtained from gene deletion studies involving the loss of the *IPMK* gene, where both InsP_5_ and InsP_6_ decreased to undetectable levels.^20^ However, genetic removal of the IPMK gene requires several weeks of selection to obtain the genetic knockout, whereas compound **14** treatment was for only 48 h, providing a potential explanation for differences between genetic and chemical results. We note that while InsP_4_ can be generated by other enzymes in mammalian cells, the only known source of InsP_5_ in mammalian cells is IPMK. We also note the large difference between *in vitro* IC_50_ on the pure, recombinant kinase vs. the effect in cells. Together, these data suggest that 48 h of treatment with compound **14** not only inhibited human U251-MG glioblastoma cell growth but also induced a novel pattern of changes to cellular inositol phosphate levels. InsP_5_, a direct product of IPMK kinase activity, was decreased, while there was no detectable effect on InsP_6_. We next attempted a longer treatment with compound **14** (72 h) in order to detect IP7 levels (Figure 7A). This showed that compound **14** did not change IP_6_ or InsP_7_ levels even after 72 h (Figure 7B), while InsP_5_ levels maintained a similar robust decrease as seen at 48 h of treatment, and InsP_4_ levels again rose slightly (Figure 7C). To the best of our knowledge, this unique metabolic signature has not been observed in any IPMK gene knockout studies performed in human cells.

**Figure 6:**
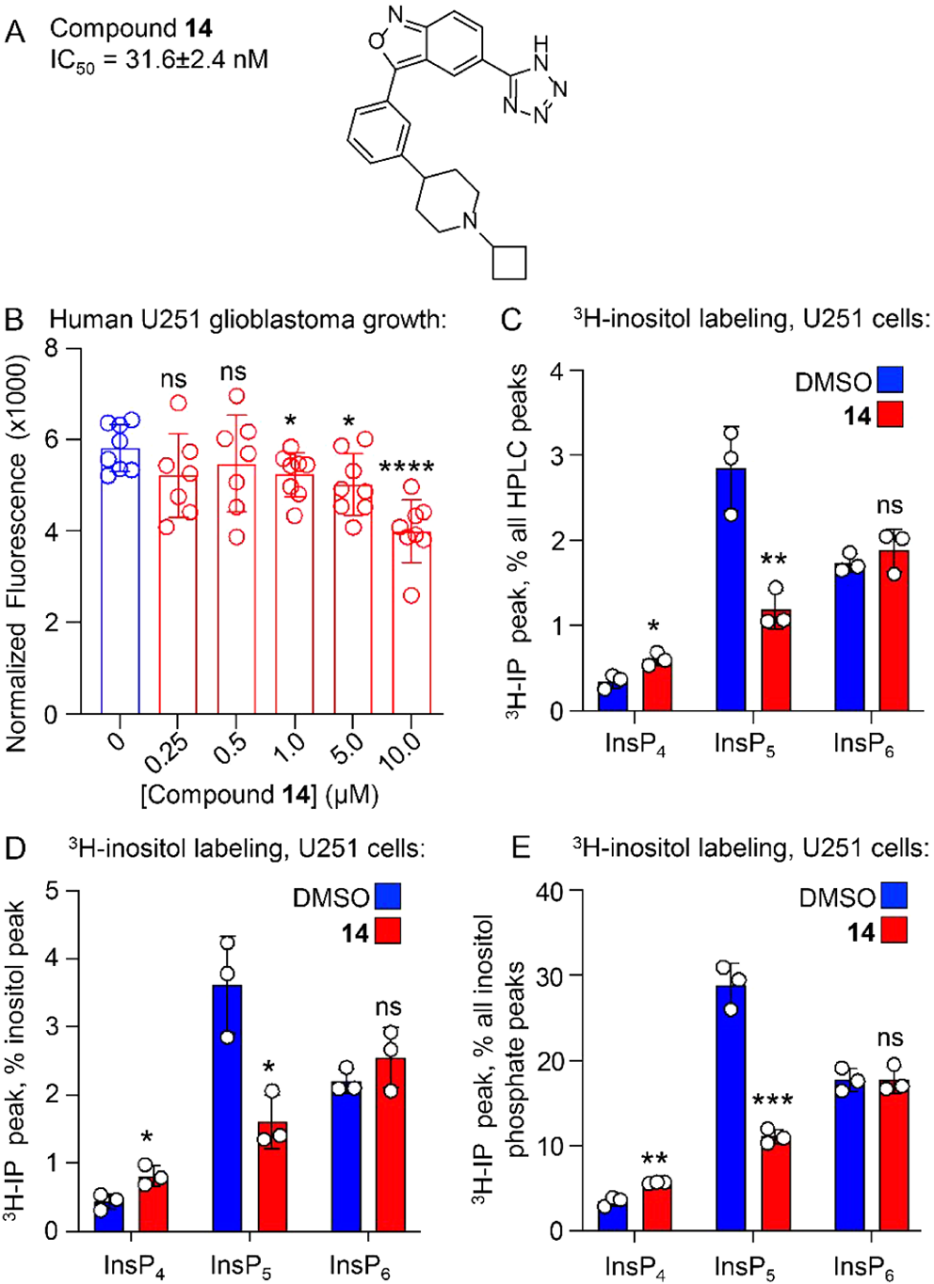
Compound **14** inhibits cell growth and InsP_5_ abundance in human U251-MG glioblastoma cells. (A) Chemical structure of 14 and measured IC_50_ for the IPMK *in vitro* kinase assay. Error represents the standard error from 3 independent assays. (B) CyQuant cellular proliferation assay of 10,000 human U251-MG glioblastoma cells seeded per well, treated for 48 h with DMSO vehicle or 10 μM **14** (*n* = 8, ****p_adj_ < 0.0001 by one-way ANOVA, Dunnett’s corrected; *p < 0.05 by unpaired *t* test). (C) HPLC analysis of ^3^H-inositol phosphates from U251-MG cells treated with 10 μM compound **14** (red bars) or DMSO (blue bars) and metabolically labeled with ^3^H-inositol for 48 h, expressed as a percentage of all the peaks detectable from the HPLC, *p < 0.05, **p < 0.01, ***p < 0.001 by unpaired *t* test compared to DMSO-treated control for all remaining panels, *n* = 3. (D) Same as (C) but expressed as a percentage of the inositol HPLC peak, *n* = 3. (E) Same as C but expressed as a percentage of all the inositol phosphate peaks combined from the HPLC, *n* = 3.

**Figure 7:**
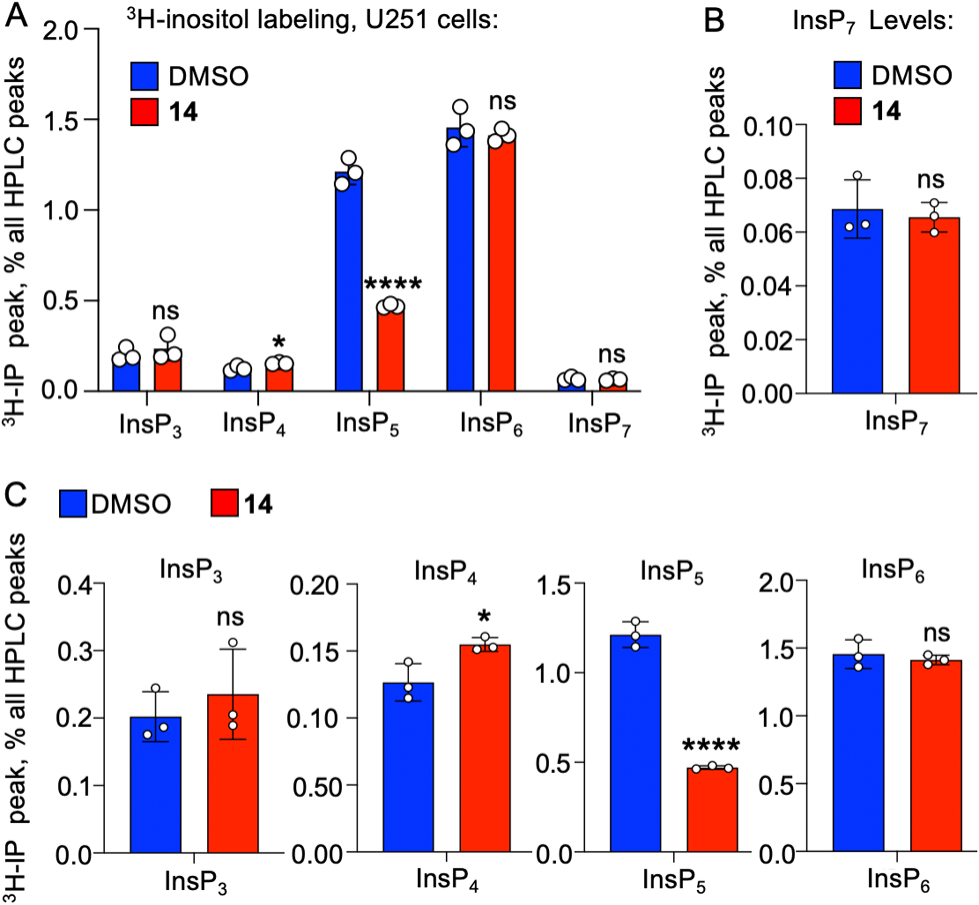
Compound **14** does not decrease IP6 or InsP_7_ levels in human U251-MG glioblastoma cells after 72 h of treatment. (A) Following 72 h of 10 μM **14** treatment, ^3^H-inositol metabolically labeled U251-MG cells were analyzed for the indicated inositol phosphates by HPLC, expressed as a percentage of all ^3^H incorporated, *p < 0.05, ****p < 0.0001 by unpaired *t* test compared to the DMSO control, for all panels *n* = 3. (B) Same as (A) but only showing the InsP_7_ peak for comparison between DMSO and **14** treatment. (C) Same as (A) but only showing each indicated inositol phosphate peak for comparison between DMSO and **14** treatment.

### Human Kinome Profiling of Compound **14** Reveals Several Off-Targets at 1 μM Concentration

To begin analyzing potential off-targets of compound **14**, kinome profiling of 30 ^20^ different human kinases was performed at 1 μM compound **14** (Figure 8A,B), a concentration >30-fold higher than its IPMK IC_50_ (Figure 4J). This 30-kinase panel was selected to represent a broad survey of kinase families, including tyrosine and serine/threonine kinases. Compound **14** (1 μM) had ≥75% inhibition of four kinases (DAPK1, DYRK1B, PDGFR, and KDR, Figure 8A,B), or 13% of all 30 kinases tested. For comparison, a comprehensive study of 369 kinase inhibitors also tested at 1 μM each, showed that 122 compounds inhibited at least four kinases greater than 75%, representing 33% of all 369 inhibitors tested.^35^ More specifically examining inhibition by these 369 inhibitors on the four kinases mentioned above (DAPK1, DYRK1B, PDGFR, and KDR), DAPK1 was ≥75% inhibited by zero (none) of the 369 inhibitors tested, DYRK1B was >75% inhibited by 13 of 369 inhibitors (4%), KDR was ≥75% inhibited by 18 of the 369 inhibitors (5%) and PDGFR was ≥75% inhibited by 42 of 369 inhibitors (11%).^35^ Compound **14** inhibition data were also mapped onto a dendrogram of the human kinome (Figure 8C), suggesting that the tyrosine kinase subgroup contains the most off-target kinases. These data suggest that 1 μM compound **14** will inhibit several other kinases in addition to IPMK, and caution must be employed when interpreting biological results as with any new kinase inhibitor development effort. However, identifying these potential off-target kinases permits future optimization that can increase on-target selectivity toward IPMK.

**Figure 8:**
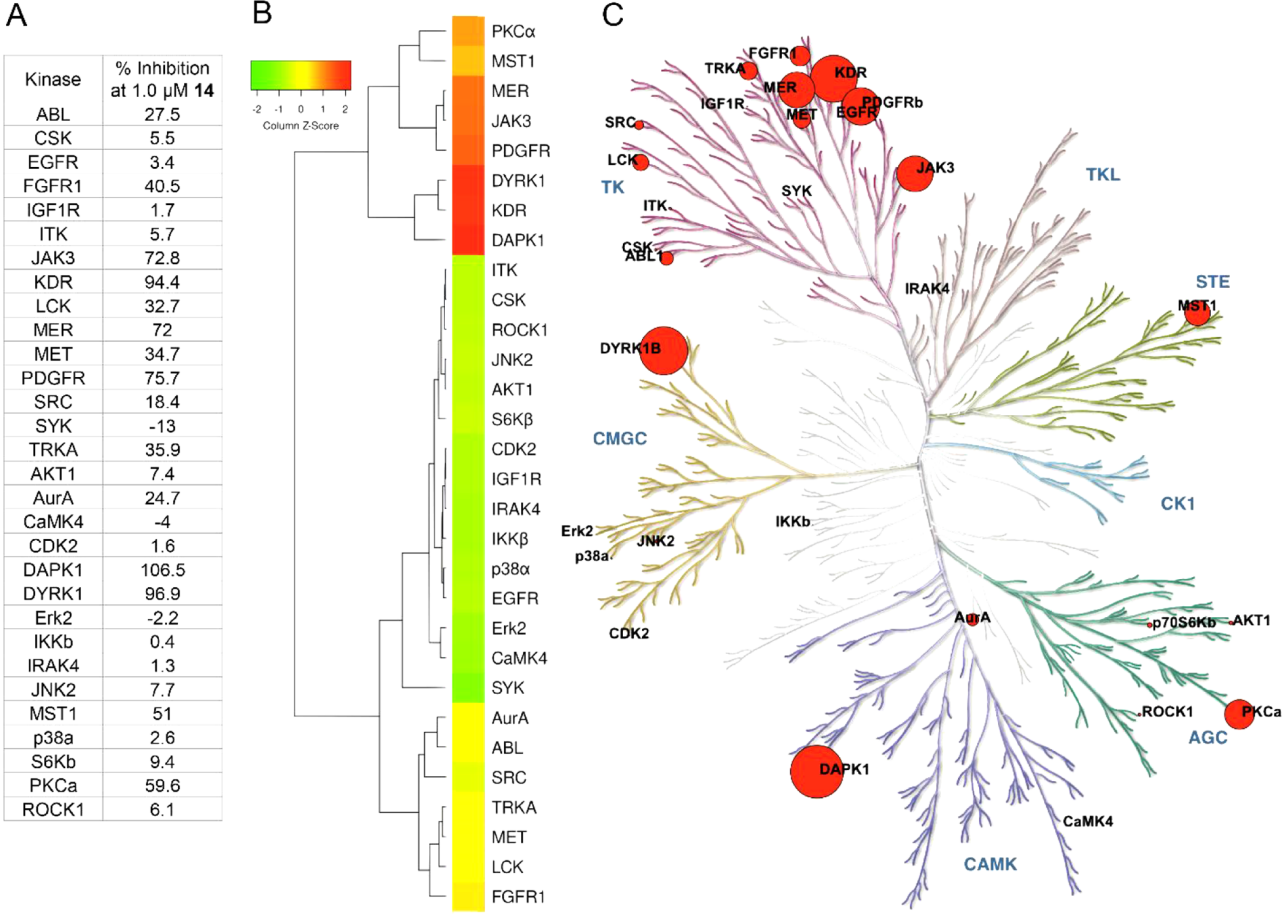
Human kinome profiling reveals that 1.0 μM **14** inhibits several kinases in addition to IPMK. IPMK is not represented within this screening panel of 30 kinases tested for **14** activity, recall **14** has maximum IPMK inhibition at 100 nM (see Figure 4J), whereas 1.0 μM **14** was tested here. (A) Alphabetically arranged table of results from human kinome profiling of 30 indicated kinases using 1.0 μM **14**, represented as percentage kinase inhibition, the kinase product ratio was calculated from the peak heights of the product and substrate peptides (P/(P + S)), the product ratio values for a control DMSO reaction set as 0% inhibition, and the “no-kinase added” background set as 100% inhibition. (B) Centroid-clustered heatmap of the inhibition data in panel (A), greater than 75% inhibition against four kinases was observed by 1.0 μM **14** (DAPK1, KDR, DYRK1, and PDGFR). (C). Phylogenetic tree of the human kinome with all kinases tested labeled by name at their position on the tree, inhibition by 1.0 μM **14** is represented by the size of the red dot, proportional to the inhibition data in panel (A), and tree was generated using KinMap. AGC: containing PKA, PKG, PKC families; CAMK: calcium/calmodulin-dependent protein kinase; CK1: casein kinase 1; CMGC: containing CDK, MAPK, GSK3, CLK families; STE: homologues of yeast Sterile 7, Sterile 11, Sterile 20 kinases; TK: tyrosine kinase; TKL: tyrosine kinase-like.

Together, the data presented here suggest that these new compounds have drug development potential for the eventual treatment of glioblastoma and perhaps other cancers, while providing a set of new chemical tools that can advance research investigating inositol phosphate and IPMK signaling. Importantly, these compounds now permit the examination of how IPMK kinase activity regulates basic biological functions both in cells and in living animals, without the caveats of long-term genetic knockouts, while minimizing the effects of adaptation to genetic knockouts of IPMK in cell-based and animal models of human disease.

## Discussion

This study suggests that small-molecule IPMK-selective inhibitors may have therapeutic value in slowing cancer cell growth, particularly in glioblastoma. U251-MG cells were selected to characterize the effects of the compounds on cellular function because they are PTEN-negative and because a previous study from Alfred Yung’s lab had shown that the proliferation of these cells can only be reduced by the expression of wild-type PTEN and a phosphatase-dead PTEN mutant, but only if wild-type PTEN was expressed in the nucleus. Cytoplasmic expression of PTEN had no effect on U251-MG cell growth.^24^ Attenuation of proliferation by nuclear wild-type PTEN was independent of any effects on AKT phosphorylation. This suggests that AKT activation by classic PI3-kinase signaling may play a less important role in human glioblastoma U251 cell growth. Indeed, although IPMK has been shown to have PI3-kinase activity on the phosphoinositide PI­(4,5)­P_2_ in membranes, we did not detect any changes to cytosolic AKT phosphorylation after 48 h of treatment with compound **14** (Figure S14). However, in an HEK cell line previously shown to respond to the PI3-kinase activity of IPMK, compound **14** was able to regulate nuclear receptor SF-1 target genes (Figure S15). Our data showing that several IPMK kinase inhibitors reduced U251-MG cell growth represent a pharmacological recapitulation of the slow-growth phenotype induced by overexpression of nuclear PTEN in these same human U251-MG glioblastoma cells. It is, therefore, tempting to speculate that chemical inhibitors of IPMK may be capable of uniquely targeting PTEN-negative glioblastoma, particularly in patients who have not responded well to therapies that decrease AKT phosphorylation. Indeed, further structure–activity relationship studies to improve compound **14** deserve thorough consideration as a potential future therapy in PTEN-negative glioblastoma or other brain cancers.

The purified human IPMK enzyme can generate both InsP_4_ and InsP_5_. In cells, InsP_4_ can also be generated by IP3K, but InsP_5_ production is known to occur only through IPMK in human cells. IPMK-generated InsP_5_ is the required precursor for the generation of InsP_6_ by IPK1. For this reason, when the IPMK gene was genetically removed from cells, those cells had decreased labeled InsP_5_ and concomitantly decreased labeled InsP_6_. Compound **14** decreased levels of InsP_5_ but had no effect on InsP_6_ after 48 h of treatment, a novel observation that highlights the importance of orthogonal chemical validation of genetic results. These data are consistent with compound **14** rapidly attenuating IPMK activity in the U251 cells with high specificity, which prevents label incorporation into the most direct product of IPMK kinase activity (InsP_5_).

The observed changes in gene expression, particularly those related to cell growth (e.g., mitotic spindle, MYC targets, KRAS signaling, and the Verhaak-Glioblastoma Classical gene sets) and inflammatory responses (e.g., NFKBIA target genes, viral/inflammation gene sets), are likely on-target perturbations related to IPMK’s kinase-dependent activity. Given that IPMK and inositol phosphates have established links to transcriptional regulation,^21,36,37^ these pathways represent a plausible direct consequence of the effect of compound **1** on IPMK. The viral and inflammatory gene sets from the GSEA are consistent with the role of InsP_6_ in viral capsid formation that occurs during infections.^38–41^ Further investigation into pathways not directly linked to IPMK’s established roles in transcription or inositol phosphate signaling but still significantly impacted by compound **1** could uncover off-target perturbations that can reveal new therapeutic avenues or areas where further compound specificity should be refined.

Several potential off-target kinases were identified in the kinome study, but the top three off-target kinases were DYRK1, KDR (also known as VEGFR-2) and DAPK1. Inhibition of these off-target kinases might lead to several biological responses that would be distinct from any activity the compound would have on IPMK. For example, DAPK1 inhibition might directly promote apoptosis and autophagy,^42^ perhaps driving cellular death programs, a role that would be distinct from IPMK’s primary biological roles.^42^ Inhibiting DYRK1A might be expected to impair synaptic plasticity, potentially leading to cognitive or neurological deficits, another function distinct from the known biological roles of IPMK.^43^ KDR (VEGFR-2) inhibition might suppress angiogenesis by blocking endothelial cell proliferation and migration, impairing new blood vessel formation in processes like tumor growth and wound healing, also a function that would be expected to be independent of IPMK inhibition.^44^ As the development of these IPMK kinase inhibitors progresses, it will be important to closely monitor these potential off-target activities.

It is also unclear why InsP_4_ levels in glioblastoma cells increased with compound **14** treatment yet decreased with compound **1** treatment. Here, we note that InsP_4_ levels also increase upon genetic deletion of IPMK in mammalian HT-29 cells.^20^ A reasonable hypothesis explaining this biological phenomenon is that compound **1** may decrease InsP_4_ due to off-target effects on IP3K and/or ITPK1 enzymes, which both generate InsP_4_. Further, upregulation of InsP_4_ by compound **14** could be due to compensatory biological upregulation of IP3K and/or ITPK1 and consequent increased production of Ins­(1,3,4,5)­P_4_ and Ins­(1,3,4,6)­P_4_, respectively, preventing the detection of InsP_4_ decreases due to IPMK inhibition. It is also worth noting that compounds with a carboxylic acid have been patented as inhibitors of IP6K.^45^ Disentangling the complex network of inositol phosphate biological regulation will be important in future studies, expedited by the new series of compounds reported here. Although IPMK has reported PI3-kinase activity on the phosphoinositide PI­(4,5)­P_2_ in cellular membranes and on a nuclear transcription factor-bound form of PI­(4,5)­P_2_, we did not detect any changes to cytosolic AKT phosphorylation after 48 h of treatment with compound **14** (Figure S14), however, we did detect changes to nuclear receptor SF-1 target genes upon treatment with compound **14** (Figure S15). Clinically, these studies also put forth the possibility of inhibiting IPMK as a therapy in PTEN-negative glioblastoma multiformes, given the favorable PK profiles of this series of kinase inhibitors. Together, these studies introduce the complete synthesis and initial biological characterization of a novel series of IPMK-directed inhibitors, which now form the basis for further medicinal chemistry optimization for eventual movement into the clinic.

## Conclusions

We identified compound **1** (UNC7437) as an IPMK inhibitor built on our lead optimization efforts to develop IP6K1 inhibitors. To remove the liability to be a potential Michael acceptor and the low permeability of acids, extensive optimization of the series was performed, leading to a more drug-like and selective IPMK inhibitor **14** (UNC9750). Compound **14** showed improved pharmacokinetic properties and effectively inhibited the cellular accumulation of InsP_5_, a direct product of IPMK kinase activity. Importantly, from a biological perspective, compound **14** induced a novel InsP_5_ metabolic signature that has not been observed in genetic knockout/complementation models of IPMK, providing new biological insights into inositol phosphate metabolism and signaling.

## Experimental Section

### Synthesis of Compounds

Microwave reactions were carried out using a CEM Discover-S reactor equipped with a vertically focused IR external temperature sensor and an Explorer 72 autosampler. The dynamic mode was used to set up the desired temperature and hold time with the following fixed parameters: PreStirring, 1 min; Pressure, 200 psi; Power, 200 W; PowerMax, off; Stirring, high. Sonication was carried out on a Branson 3510 Ultrasonic Cell. Centrifugation was carried out on an Eppendorf Centrifuge 5418. Flash chromatography was carried out on a Teledyne ISCO CombiFlash R_f_ 200 system with prepacked silica gel disposable columns or prepacked reverse-phase C18 columns. Analytical thin-layer chromatography (TLC) was performed with silica gel 60 F_254_, 0.25 mm precoated TLC plates. TLC plates were visualized using UV_254_ light and phosphomolybdic acid with charring. All ^1^H NMR spectra were obtained with a 400 or 500 MHz spectrometer using CDCl_3_ (7.26 ppm), DMSO-*d*_6_ (2.50 ppm, quintet), or CD_3_OD (3.31 ppm, quintet) as an internal reference. Signals are reported as m (multiplet), s (singlet), d (doublet), t (triplet), q (quartet), and bs (broad singlet); coupling constants are reported in hertz (Hz). ^13^C NMR spectra were obtained with a 100 or 125 MHz spectrometer using CDCl_3_ (77.2 ppm, triplet), DMSO-*d*_6_ (39.5 ppm, septet), or CD_3_OD (49.3 ppm, septet) as the internal standard. LC/MS was performed using an analytical instrument with the UV detector set to 220, 254, and 280 nm and a single quadrupole mass spectrometer using electrospray ionization (ESI) source. Samples were injected (2 μL) onto a 4.6 mm × 50 mm, 1.8 μm, C18 column at room temperature. A linear gradient from 10% to 100% B (MeOH + 0.1% acetic acid) in 5.0 min was followed by pumping 100% B for an additional 2 or 4 min with A being H_2_O + 0.1% acetic acid. The flow rate was 1.0 mL/min. Purity is >95% for all final compounds determined by LC-MS. Analytical HPLC was performed with prominence diode array detector (SPD-M20A). Samples were injected onto a 3.6 μm PEPTIDE XB-C18 100 Å, 150 × 4.6 mm LC column at room temperature. The flow rate was 1.0 mL/min. Various linear gradients were used with A being H_2_O + 0.1% TFA and B being acetonitrile +0.1% TFA.

#### (*E*)-3-(3-(3,5-Dimethylphenyl)­benzo­[*c*]­isoxazol-5-yl)­acrylic Acid (**1** ^25^

NaOH pellets were granulated (300 mg, 5.00 mmol) and added to *i*-PrOH (7.5 mL). The mixture was placed in the ultrasonic cell until NaOH was totally suspended, then was added 4-nitrobenzaldehyde glycol acetal (293 mg, 1.50 mmol). The reaction mixture was stirred until the solid dissolved and then was added 2-(3,5-dimethylphenyl)­acetonitrile (544 mg, 3.75 mmol). The reaction mixture was stirred at rt and monitored by TLC. Upon completion of the reaction, a light-yellow solid precipitated. The solid was filtered and washed with saturated Na_2_S_2_O_3_ solution, water, and *i*-PrOH.

Without further purification, the solid was added to a mixture of MeCN (6.0 mL) and water (1.5 mL) and then treated with TFA (300 μL, 3.92 mmol). The reaction mixture was stirred at rt for 3 h, quenched with a saturated NaHCO_3_ solution, and extracted with CH_2_Cl_2_. The organic phase was dried (Na_2_SO_4_) and concentrated under reduced pressure. The residue was purified by an ISCO silica gel column (hexane/EtOAc gradient) to afford 3-(3,5-dimethylphenyl)­benzo­[*c*]­isoxazole-5-carbaldehyde as a yellow solid (189 mg, 0.755 mmol, 50%). ^1^H NMR (400 MHz, CDCl_3_) δ 10.01 (s, 1H), 8.38 (s, 1H), 7.83 (dd, *J* = 9.3, 1.3 Hz, 1H), 7.70–7.63 (m, 3H), 7.22 (dd, *J* = 1.6, 0.8 Hz, 1H), 2.47 (s, 6H); MS (ESI) for [M + H]^+^ (C_16_H_14_NO_2_^+^): calcd. *m*/*z* 252.10; found *m*/*z* 252.10; LC-MS: 98% purity.

To a solution of 3-(3,5-dimethylphenyl)­benzo­[*c*]­isoxazole-5-carbaldehyde (189 mg, 0.751 mmol) in CH_2_Cl_2_ (7.5 mL) was added ethyl 2-(triphenylphosphoranylidene)­acetate (274 mg, 0.788 mmol). The resulting solution was stirred at rt for 2 h. The solvent was concentrated under reduced pressure. The residue was purified by an ISCO silica gel column (hexane/EtOAc gradient) to afford compound **5** as a yellow solid (237 mg, 0.738 mmol, 98%) with an *E*/*Z* of 97:3 (LC-MS). MS (ESI) for [M + H]^+^ (C_20_H_20_NO_3_^+^): calcd. *m*/*z* 322.14; found *m*/*z* 322.10; LC-MS: 97% purity.

To a solution of **5** (237 mg, 0.738 mmol) in MeCN (5.0 mL) was added an aqueous solution of NaOH (88.6 mg, 2.21 mmol) in water (2.4 mL). The resulting suspension was heated at 60 °C for 1.5 h, cooled to rt, and treated with 1.0 M HCl to adjust the pH to 1. The mixture was added to a small amount of MeOH and refluxed for 1 h to remove the *Z*-isomer. The precipitate was filtered and washed with a small amount of MeOH and water to afford the title compound **1** (183 mg, 0.625 mmol, 85%) as a yellow solid. ^1^H NMR (400 MHz, DMSO-*d*_6_) δ 8.46 (s, 1H), 7.86 (d, *J* = 9.4 Hz, 1H), 7.84–7.76 (m, 3H), 7.67 (d, *J* = 9.4 Hz, 1H), 7.24 (s, 1H), 6.61 (d, *J* = 16.0 Hz, 1H), 2.42 (s, 6H); ^13^C NMR (101 MHz, DMSO-*d*_6_) δ 167.72, 165.40, 157.19, 143.38, 139.07, 132.67, 131.48, 129.25, 127.00, 124.55, 124.20, 119.44, 115.63, 113.91, 20.84; MS (ESI) for [M + H]^+^ (C_18_H_16_NO_3_^+^): calcd. *m*/*z* 294.11; found *m*/*z* 294.10; LC-MS: 99% purity.

#### (±)-*trans*-2-(3-(3,5-Dimethylphenyl)­benzo­[*c*]­isoxazol-5-yl)­cyclopropane-1-carboxylic acid (**2**)

To a solution of trimethylsulfoxonium iodide (139 mg, 0.630 mmol) in anhydrous DMSO (0.50 mL) was added NaH (25.2 mg, 0.630 mmol, 60 wt % suspension in mineral oil) at 0 °C. After stirring at 0 °C for 20 min, a solution of **5** (96.4 mg, 0.300 mmol) in anhydrous DMSO (1.0 mL) was added dropwise. The reaction mixture was stirred at rt for 24 h and poured into a sat. NH_4_Cl solution. The mixture was extracted with EtOAc (3×), dried (Na_2_SO_4_), and concentrated under reduced pressure. The residue was purified by an ISCO silica gel column (hexane/EtOAc gradient) to afford ethyl *trans*-2-(3-(3,5-dimethylphenyl)­benzo­[*c*]­isoxazol-5-yl)­cyclopropane-1-carboxylate as a yellow solid (35.3 mg, 0.105 mmol, 35%). ^1^H NMR (400 MHz, CDCl_3_) δ 7.62–7.52 (m, 4H), 7.14 (d, *J* = 2.0 Hz, 1H), 7.02 (dd, *J* = 9.3, 1.6 Hz, 1H), 4.20 (q, *J* = 7.1 Hz, 2H), 2.65–2.57 (m, 1H), 2.44 (s, 6H), 1.96 (ddd, *J* = 8.5, 5.3, 4.2 Hz, 1H), 1.64 (dt, *J* = 9.1, 5.0 Hz, 1H), 1.43–1.36 (m, 1H), 1.30 (t, *J* = 7.1 Hz, 3H); MS (ESI) for [M + H]^+^ (C_21_H_22_NO_3_^+^): calcd. *m*/*z* 336.16; found *m*/*z* 336.20; LC-MS: 95% purity.

To a solution of ethyl *trans*-2-(3-(3,5-dimethylphenyl)­benzo­[*c*]­isoxazol-5-yl)­cyclopropane-1-carboxylate (35.3 mg, 0.105 mmol) in MeCN (0.75 mL) was added a solution of NaOH (12.6 mg, 0.12 mmol) in water (0.25 mL). The resulting suspension was heated at 60 °C for 3.0 h, cooled to rt, acidified with 1.0 M HCl solution to pH 1, and extracted with EtOAc (3×). The combined organic phase was washed with brine, dried (Na_2_SO_4_), and concentrated under reduced pressure. The residue was purified by a reverse-phase ISCO silica gel column (MeCN/H_2_O gradient) to afford the title compound **2** (21.6 mg, 70.3 μmol, 67%) as a pale brown solid. ^1^H NMR (400 MHz, CDCl_3_) δ 7.63–7.52 (m, 4H), 7.14 (dt, *J* = 2.0, 1.0 Hz, 1H), 7.04 (dd, *J* = 9.1, 1.6 Hz, 1H), 2.75–2.66 (m, 1H), 2.45 (s, 6H), 1.98 (ddd, *J* = 8.4, 5.4, 4.2 Hz, 1H), 1.72 (dt, *J* = 9.6, 5.0 Hz, 1H), 1.49 (ddd, *J* = 8.4, 6.7, 4.8 Hz, 1H). ^13^C NMR (101 MHz, CDCl_3_) δ 179.00, 164.60, 157.51, 139.13, 135.52, 132.28, 130.26, 128.32, 124.52, 117.70, 116.19, 114.35, 27.47, 23.14, 21.58, 16.87; MS (ESI) for [M + H]^+^ (C_19_H_18_NO_3_^+^): calcd. *m*/*z* 308.13; found *m*/*z* 308.10; LC-MS: 97% purity.

#### 3-(3,5-Dimethylphenyl)-5-(1*H*-tetrazol-5-yl)­benzo­[*c*]­isoxazole (**4**)

(General procedure A) NaOH pellets were granulated (80.0 mg, 2.00 mmol) and added to *i*-PrOH (1.0 mL). The mixture was placed in the ultrasonic cell until NaOH was totally suspended; then 5-(4-nitrophenyl)-1*H*-tetrazole was added (38.2 mg, 0.200 mmol). The reaction mixture was stirred until the solid dissolved, then 2-(3,5-dimethylphenyl)­acetonitrile (87.1 mg, 0.600 mmol) was added. The reaction was stirred at rt and monitored by TLC. Upon completion of the reaction, the suspension was acidified by glacial acetic acid to pH 5. A light-yellow precipitate was collected by filtration, washed with a small amount of EtOAc, and purified by a reverse ISCO silica gel column to afford **4** as a yellow solid (15.1 mg, 51.8 μmol, 26%). ^1^H NMR (400 MHz, DMSO-*d*_6_) δ 8.72 (t, *J* = 1.3 Hz, 1H), 8.05 (dd, *J* = 9.3, 1.4 Hz, 1H), 7.89 (dd, *J* = 9.3, 1.0 Hz, 1H), 7.76 (d, *J* = 1.6 Hz, 2H), 7.29 (d, *J* = 1.8 Hz, 1H), 2.44 (s, 6H). ^13^C NMR (101 MHz, DMSO-*d*_6_) δ: 166.39, 156.93, 154.82, 139.12, 132.94, 129.59, 126.82, 124.32, 121.15, 120.77, 116.47, 113.40, 20.89. MS (ESI) for [M + H]^+^ (C_16_H_14_N_5_O^+^): *m*/*z* 292.12; found *m*/*z* 292.10; LC-MS: 97% purity.

#### 3-Phenyl-5-(1*H*-tetrazol-5-yl)­benzo­[*c*]­isoxazole (**6**)

The title compound **6** (38.7 mg, 0.147 mmol, 74%) was prepared according to general procedure A from 5-(4-nitrophenyl)-1*H*-tetrazole (38.2 mg, 0.200 mmol) and benzyl cyanide (57.7 μL, 0.500 mmol) as a yellow solid. ^1^H NMR (400 MHz, DMSO-*d*_6_) δ 8.76 (t, *J* = 1.1 Hz, 1H), 8.20–8.12 (m, 2H), 8.06 (dd, *J* = 9.3, 1.5 Hz, 1H), 7.90 (dd, *J* = 9.3, 1.0 Hz, 1H), 7.77–7.61 (m, 3H); ^13^C NMR (101 MHz, DMSO-*d*_6_) δ 166.08, 156.96, 154.95, 131.43, 129.77, 129.59, 126.90, 126.76, 121.00, 120.97, 116.51, 113.56; MS (ESI) for [M + H]^+^ (C_14_H_10_N_5_O^+^): calcd. *m*/*z* 264.09; found *m*/*z* 264.10; LC-MS: 99% purity.

#### 5-(1*H*-Tetrazol-5-yl)-3-(4-(trifluoromethyl)­phenyl)­benzo­[*c*]­isoxazole (**7**)

The title compound **7** (17.4 mg, 52.5 μmol, 26%) was prepared according to general procedure A from 5-(4-nitrophenyl)-1*H*-tetrazole (38.2 mg, 0.200 mmol) and 4-trifluoromethylphenylacetonitrile (60.2 μL, 0.400 mmol) as a yellow solid. ^1^H NMR (400 MHz, DMSO-*d*_6_) δ 8.78 (d, *J* = 1.3 Hz, 1H), 8.36 (d, *J* = 8.2 Hz, 2H), 8.11–7.99 (m, 3H), 7.96 (dd, *J* = 9.3, 1.0 Hz, 1H). ^13^C NMR (101 MHz, DMSO-*d*_6_) δ 164.30, 157.05, 130.90, 130.58, 130.38, 129.76, 127.52, 126.58 (q, *J* = 3.9 Hz), 123.80 (q, *J* = 274 Hz), 121.89, 120.54, 116.79, 114.49. MS (ESI) for [M + H]^+^ (C_15_H_9_F_3_N_5_O^+^): calcd. *m*/*z* 332.08; found *m*/*z* 332.10; LC-MS: 99% purity.

#### 5-(1*H*-Tetrazol-5-yl)-3-(4-(trifluoromethoxy)­phenyl)­benzo­[*c*]­isoxazole (**8**)

The title compound **8** (53.5 mg, 0.154 mmol, 77%) was prepared according to general procedure A from 5-(4-nitrophenyl)-1*H*-tetrazole (38.2 mg, 0.200 mmol) and 4-trifluoromethoxyphenylacetonitrile (94.3 μL, 0.600 mmol) as a yellow solid. ^1^H NMR (400 MHz, DMSO-*d*_6_) δ 8.75 (d, *J* = 1.3 Hz, 1H), 8.34–8.25 (m, 2H), 8.08 (dd, *J* = 9.3, 1.4 Hz, 1H), 7.94 (dd, *J* = 9.4, 1.0 Hz, 1H), 7.76–7.66 (m, 2H). ^13^C NMR (101 MHz, DMSO-*d*_6_) δ 164.66, 157.01, 155.24, 150.00 (q, *J* = 1.7 Hz), 129.80, 129.07, 126.04, 122.18, 121.69, 120.56, 120.01 (q, *J* = 259 Hz), 116.55, 113.92. MS (ESI) for [M + H]^+^ (C_15_H_9_F_3_N_5_O_2_^+^): calcd. *m*/*z* 348.07; found *m*/*z* 348.05; LC-MS: 97% purity.

#### 3-(4-Phenoxyphenyl)-5-(1*H*-tetrazol-5-yl)­benzo­[*c*]­isoxazole (**9**)

The title compound **9** (32.0 mg, 90.0 μmol, 45%) was prepared according to general procedure A from 5-(4-nitrophenyl)-1*H*-tetrazole (38.2 mg, 0.200 mmol) and 4-phenoxyphenylacetonitrile (105 mg, 0.500 mmol) as a yellow solid. ^1^H NMR (400 MHz, DMSO-*d*_6_) 8.44 (s, 1H), 8.18 (dd, *J* = 9.3, 1.3 Hz, 1H), 8.15–8.06 (m, 2H), 7.72–7.65 (m, 1H), 7.52–7.44 (m, 2H), 7.26 (dd, *J* = 8.4, 2.1 Hz, 3H), 7.18 (dd, *J* = 7.7, 1.5 Hz, 2H); MS (ESI) for [M + H]^+^ (C_20_H_14_N_5_O_2_^+^): calcd. *m*/*z* 356.11; found *m*/*z* 356.10; LC-MS: 95% purity.

#### *N*-(4-(5-(1*H*-Tetrazol-5-yl)­benzo­[*c*]­isoxazol-3-yl)­phenyl)­cyclopropanecarboxamide (**10**)

(General procedure B) To a solution of 2-(4-aminophenyl)­acetonitrile (200 mg, 1.51 mmol) in DMF (3.0 mL) was added cyclopropanecarboxylic acid (122 μL, 1.51 mmol), DIPEA (527 μL, 3.03 mmol), and HATU (633 mg, 1.66 mmol). The resulting mixture was stirred at rt for 1 h and quenched with water (2.5 mL). The product was crushed and isolated by centrifugation. The crude product was washed with water (2×) and dried under lyophilization to afford *N*-(4-(cyanomethyl)­phenyl)­cyclopropanecarboxamide as an off-white solid (248 mg, 1.24 mmol, 82%). ^1^H NMR (400 MHz, CDCl_3_) δ 7.53 (d, *J* = 8.3 Hz, 2H), 7.41 (s, 1H), 7.27 (d, *J* = 8.1 Hz, 2H), 3.71 (s, 2H), 1.50 (tt, *J* = 8.0, 4.5 Hz, 1H), 1.12 – 1.06 (m, 2H), 0.87 (dt, *J* = 7.8, 3.5 Hz, 2H); MS (ESI) for [M + H]^+^ (C_12_H_13_N_2_O^+^): calcd. *m*/*z* 201.10; found *m*/*z* 201.10; LC-MS: 95% purity.

To a mixture of 5-(4-nitrophenyl)-1*H*-tetrazole (57.3 mg, 0.300 mmol) and *N*-(4-(cyanomethyl)­phenyl)­cyclopropanecarboxamide (50.1 mg, 0.250 mmol) in 1-butyl-3-methylimidazolium tetrafluoroborate ([Bmim]­BF_4_) (2.5 mL) was added triazabicyclodecene (TBD, 348 mg, 2.50 mmol). The reaction mixture was stirred at rt and monitored by LCMS. Upon completion of the reaction, the mixture was acidified by HOAc to pH 5, quenched with brine, and extracted with EtOAc (3×). The combined organic phase was washed with brine, dried (Na_2_SO_4_), and concentrated under reduced pressure. The residue was purified by a reverse-phase ISCO silica gel column (MeCN/H_2_O gradient) to afford the title compound **10** (34.2 mg, 98.7 μmol, 39%) as a yellow solid. ^1^H NMR (400 MHz, DMSO-*d*_6_) δ 10.63 (s, 1H), 8.77 (s, 1H), 8.18–8.09 (m, 2H), 8.04 (dd, *J* = 9.3, 1.4 Hz, 1H), 7.96–7.89 (m, 2H), 7.87 (d, *J* = 9.4 Hz, 1H), 1.89–1.80 (m, 1H), 0.93–0.82 (m, 4H); ^13^C NMR (126 MHz, DMSO-*d*_6_) δ 172.35, 166.09, 156.94, 142.14, 133.51, 129.51, 127.66, 121.45, 121.25, 119.44, 116.34, 112.93, 40.02, 14.83, 7.68; MS (ESI) for [M + H]^+^ (C_18_H_15_N_6_O_2_^+^): calcd. *m*/*z* 347.13; found *m*/*z* 347.10; LC-MS: 98% purity.

#### *N*-(3-(5-(1*H*-Tetrazol-5-yl)­benzo­[*c*]­isoxazol-3-yl)­phenyl)­cyclopropanecarboxamide (**11**)

The title compound **11** (28.6 mg, 82.6 μmol, 33%) was prepared according to general procedure B from 2-(3-aminophenyl)­acetonitrile (134 μL, 1.13 mmol) and cyclopropanecarboxylic acid (90.4 μL, 1.13 mmol) as a yellow solid. ^1^H NMR (400 MHz, DMSO-*d*_6_) δ 10.57 (s, 1H), 8.72 (s, 1H), 8.42 (s, 1H), 8.04 (d, *J* = 9.3 Hz, 1H), 7.90 (d, *J* = 9.4 Hz, 1H), 7.83 (d, *J* = 8.1 Hz, 2H), 7.62 (t, *J* = 8.0 Hz, 1H), 1.83 (t, *J* = 6.2 Hz, 1H), 0.91–0.84 (m, 4H); ^13^C NMR (214 MHz, DMSO- *d*_6_) δ 172.37, 166.10, 157.09, 155.41, 140.55, 130.41, 129.74, 127.33, 121.72, 121.33, 121.30, 120.87, 116.73, 116.68, 113.71, 14.83, 7.59; MS (ESI) for [M + H]^+^ (C_18_H_15_N_6_O_2_^+^): calcd. *m*/*z* 347.13; found *m*/*z* 347.10; LC-MS: 98% purity.

#### 3-(3-(Piperidin-4-yloxy)­phenyl)-5-(1*H*-tetrazol-5-yl)­benzo­[*c*]­isoxazole (**12**)

To a solution of 2-(3-hydroxyphenyl)­acetonitrile (2.00 g, 15.0 mmol), *tert*-butyl 4-hydroxypiperidine-1-carboxylate (3.33 g, 16.5 mmol), and PPh_3_ (4.33 g, 16.5 mmol) in anhydrous THF (15 mL) was added diisopropyl azodicarboxylate (DIAD, 3.28 mL, 16.5 mmol) at 0 °C. The resulting mixture was stirred at rt for 24 h. Upon completion, the reaction mixture was passed through a short pad of silica gel. The silica gel was then washed with a mixture of hexane and EtOAc (2:1, 150 mL). The organic phase was concentrated to about 50 mL and washed with a 1.0 M NaOH solution (3×) and brine. The organic layer was dried (Na_2_SO_4_) and concentrated under reduced pressure. The residue was dissolved in CH_2_Cl_2_ and loaded onto a silica gel column. The product was isolated from silica gel column chromatography as an oil, which was poured into hexane (45 mL). The mixture was sonicated until a white solid crushed out to afford a fine suspension. The precipitate was filtered and washed with hexane to afford *tert*-butyl 4-(3-(cyanomethyl)­phenoxy)­piperidine-1-carboxylate as a white solid (3.82 g, 12.1 mmol, 80%). ^1^H NMR (400 MHz, chloroform-*d*) δ 7.31–7.26 (m, 1H), 6.94–6.82 (m, 3H), 4.49 (tt, *J* = 7.1, 3.5 Hz, 1H), 3.75–3.65 (m, 2H), 3.72 (s, 2H), 3.35 (ddd, *J* = 13.5, 7.6, 3.8 Hz, 2H), 1.98–1.87 (m, 2H), 1.75 (dtd, *J* = 13.8, 7.3, 3.7 Hz, 2H), 1.47 (s, 9H); MS (ESI) for [M + Na]^+^ (C_18_H_24_N_2_O_3_Na^+^): calcd. *m*/*z* 339.17; found *m*/*z* 339.20; LC-MS: 96% purity.

NaOH pellets were granulated (0.98 g, 24 mmol) and added to 2-PrOH (25 mL). The mixture was stirred for 15 min before the addition of 5-(4-nitrophenyl)-1*H*-tetrazole (703 mg, 3.68 mmol). After the tetrazole was dissolved, *tert*-butyl 4-(3-(cyanomethyl)­phenoxy)­piperidine-1-carboxylate (775 mg, 2.45 mmol) was added. The reaction mixture was stirred at rt and monitored by LC-MS. Upon completion of the reaction, to the suspension was added HOAc pH to 5. The mixture was poured into brine and extracted by EtOAc (3×). The combined organic phase was dried (Na_2_SO_4_) and concentrated under reduced pressure. The residue was purified by reverse ISCO silica gel column (MeCN/H_2_O gradient) to afford *tert*-butyl 4-(3-(5-(1*H*-tetrazol-5-yl)­benzo­[*c*]­isoxazol-3-yl)­phenoxy)­piperidine-1-carboxylate as a yellow solid (310 mg, 0.671 mmol, 27%). ^1^H NMR (400 MHz, CD_3_OD) δ 8.72 (d, *J* = 1.6 Hz, 1H), 8.09 (d, *J* = 9.3 Hz, 1H), 7.80–7.72 (m, 2H), 7.67 (q, *J* = 1.9 Hz, 1H), 7.58 (t, *J* = 8.0 Hz, 1H), 7.23 (dd, *J* = 8.3, 2.5 Hz, 1H), 3.78 (ddd, *J* = 12.0, 7.1, 3.9 Hz, 2H), 3.41 (d, *J* = 10.7 Hz, 2H), 2.03 (dd, *J* = 13.0, 7.6 Hz, 2H), 1.76 (dtd, *J* = 12.0, 7.8, 3.8 Hz, 2H), 1.48 (s, 9H) (one proton overlaps with CD_3_OD peak); MS (ESI) for [M + H]^+^ (C_24_H_27_N_6_O_4_^+^): calcd. *m*/*z* 463.21; found *m*/*z* 463.15; LC-MS: 98% purity.

To a solution of *tert*-butyl 4-(3-(5-(1*H*-tetrazol-5-yl)­benzo­[*c*]­isoxazol-3-yl)­phenoxy)­piperidine-1-carboxylate (310 mg, 0.671 mmol) in MeOH (3.4 mL) was added 4 N HCl solution in 1,4-dioxane (1.68 mL, 6.71 mmol). The reaction mixture was stirred at rt for 3 h and concentrated under reduced pressure. The residue was sonicated in a small amount of a mixture of MeOH/H_2_O (1:1) and collected by centrifugation to afford the title compound **12** (229 mg, 0.574 mmol, 86%) as a yellow solid. ^1^H NMR (500 MHz, CD_3_OD) δ 8.68 (s, 1H), 8.03–7.96 (m, 1H), 7.77–7.70 (m, 2H), 7.67 (d, *J* = 2.2 Hz, 1H), 7.58 (t, *J* = 8.0 Hz, 1H), 7.25 (dd, *J* = 8.2, 2.5 Hz, 1H), 3.49 (ddd, *J* = 12.8, 9.1, 3.6 Hz, 2H), 3.35–3.27 (m, 2H), 2.27 (ddd, *J* = 12.9, 9.0, 3.7 Hz, 2H), 2.13 (dq, *J* = 11.0, 3.4 Hz, 2H) (one proton overlaps with CD_3_OD peak); ^13^C NMR (126 MHz, CD_3_OD) δ 166.21, 157.48, 157.27, 130.84, 129.12, 128.71, 121.34, 120.76, 119.74, 118.42, 116.17, 114.01, 113.85, 68.71, 40.52, 26.98 (one *sp* ^2^ carbon is missing); MS (ESI) for [M + H]^+^ (C_19_H_19_N_6_O_2_^+^): calcd. *m*/*z* 363.16; found *m*/*z* 363.15; LC-MS: 99% purity.

#### 3-(3-((3,5-Dichloropyridin-2-yl)­oxy)­phenyl)-5-(1*H*-tetrazol-5-yl)­benzo­[*c*]­isoxazole (**13**)

To a solution of 2-(4-hydroxyphenyl)­acetonitrile (66.6 mg, 0.500 mmol) in DMF (1.5 mL) was added 3,5-dichloro-2-fluoropyridine (87.1 mg, 0.525 mmol) and Cs_2_CO_3_ (326 mg, 1.00 mmol). The reaction mixture was stirred under microwave irradiation (100 W) at 120 °C for 15 min, quenched with brine, and extracted with EtOAc (3×). The combined organic phase was dried (Na_2_SO_4_) and concentrated under reduced pressure. The residue was purified by an ISCO silica gel column (0–20% EtOAc/hexane gradient) to afford 2-(4-((3,5-dichloropyridin-2-yl)­oxy)­phenyl)­acetonitrile (114 mg, 0.408 mmol, 82%) as an off-white solid. ^1^H NMR (400 MHz, CDCl_3_) δ 7.96 (d, *J* = 2.4 Hz, 1H), 7.78 (d, *J* = 2.4 Hz, 1H), 7.39 (d, *J* = 8.6 Hz, 2H), 7.17 (d, *J* = 8.6 Hz, 2H), 3.78 (s, 2H); MS (ESI) for [M + H]^+^ (C_13_H_9_Cl_2_N_2_O^+^): calcd. *m*/*z* 279.01; found *m*/*z* 279.05; LC-MS: 99% purity.

The title compound **13** (19.3 mg, 45.4 μmol, 18%) was prepared according to general procedure A from 5-(4-nitrophenyl)-1*H*-tetrazole (57.3 mg, 0.300 mmol) and 2-(4-((3,5-dichloropyridin-2-yl)­oxy)­phenyl)­acetonitrile (69.8 mg, 0.250 mmol) as a yellow solid. ^1^H NMR (400 MHz, DMSO-*d*_6_) δ 8.77 (s, 1H), 8.39 (d, *J* = 2.3 Hz, 1H), 8.21 (d, *J* = 2.3 Hz, 1H), 8.07 (dd, *J* = 9.3, 1.5 Hz, 2H), 8.00 (t, *J* = 2.0 Hz, 1H), 7.93 (d, *J* = 9.4 Hz, 1H), 7.77 (t, *J* = 8.0 Hz, 1H), 7.49 (dd, *J* = 8.1, 2.4 Hz, 1H); ^13^C NMR (101 MHz, DMSO-*d*_6_) δ 165.13, 157.01, 156.92, 154.89, 153.90, 143.77, 139.49, 131.38, 129.73, 128.27, 125.63, 124.40, 123.77, 121.24, 120.94, 119.57, 118.79, 116.60, 113.87; MS (ESI) for [M + H]^+^ (C_19_H_11_Cl_2_N_6_O_2_^+^): calcd. *m*/*z* 425.03; found *m*/*z* 425.00; LC-MS: 96% purity.

#### 3-(3-(1-Cyclobutylpiperidin-4-yl)­phenyl)-5-(1*H*-tetrazol-5-yl)­benzo­[*c*]­isoxazole (**14**)

To a solution of 2-(3-bromophenyl)­acetonitrile (600 mg, 3.06 mmol) and *tert*-butyl 4-(4,4,5,5-tetramethyl-1,3,2-dioxaborolan-2-yl)-3,6-dihydropyridine-1­(2*H*)-carboxylate (994 mg, 3.21 mmol) in 1,4-dioxane (4.2 mL) and water (1.4 mL) was added K_2_CO_3_ (846 mg, 6.12 mmol). The mixture was degassed and refilled with nitrogen for three cycles. Then Pd­(PPh_3_)_4_ (35.4 mg, 30.6 μmol) was added. The reaction mixture was heated under nitrogen at 90 °C for 18 h, quenched with brine, and extracted with EtOAc (3×). The combined organic phase was washed with brine, dried (Na_2_SO_4_) and concentrated under reduced pressure.

To the crude product in MeOH (10 mL) was added Pd/C (49 mg, 0.46 mmol). The reaction mixture was stirred under a hydrogen atmosphere at 40 °C for 12 h and then filtered through Celite. The solvent was removed under reduced pressure, and the residue was purified by an ISCO silica gel column (MeCN/H_2_O gradient) to afford *tert*-butyl 4-(3-(cyanomethyl)­phenyl)­piperidine-1-carboxylate (751 mg, 2.50 mmol, 82%) as a yellow foam. ^1^H NMR (400 MHz, CDCl_3_) δ 7.35–7.28 (m, 1H), 7.20–7.12 (m, 3H), 4.24 (s, 2H), 3.73 (s, 2H), 2.79 (t, *J* = 12.4 Hz, 2H), 2.65 (tt, *J* = 12.1, 3.5 Hz, 1H), 1.81 (d, *J* = 13.3 Hz, 2H), 1.68–1.53 (m, 2H), 1.48 (s, 9H); MS (ESI) for [M + H]^+^ (C_18_H_25_N_2_O_2_^+^): calcd. *m*/*z* 301.40; found *m*/*z* 301.40; LC-MS: 98% purity.

NaOH pellets were granulated (672 mg, 16.64 mmol) and added to *i*-PrOH (16 mL). The mixture was stirred for 15 min before the addition of 5-(4-nitrophenyl)-1*H*-tetrazole (382 mg, 1.99 mmol). After the tetrazole dissolved, *tert*-butyl 4-(3-(cyanomethyl)­phenyl)­piperidine-1-carboxylate (500 mg, 1.66 mmol) was added. The reaction mixture was stirred at rt and monitored by TLC. Upon completion of the reaction, the suspension was diluted with MeOH and acidified with HOAc to pH 5. The reaction mixture was diluted with EtOAc. The organic phase was washed with water and brine, dried (Na_2_SO_4_), and concentrated. The residue was purified by a reverse ISCO silica gel column (MeCN/H_2_O gradient) to afford *tert*-butyl 4-(3-(5-(1*H*-tetrazol-5-yl)­benzo­[*c*]­isoxazol-3-yl)­phenoxy)­piperidine-1-carboxylate as a yellow solid (456 mg, 1.02 mmol, 61%). ^1^H NMR (400 MHz, CD_3_OD) δ 8.67 (s, 1H), 7.99 (dd, *J* = 9.4, 1.2 Hz, 1H), 7.96 – 7.89 (m, 2H), 7.72 (d, *J* = 9.4 Hz, 1H), 7.56 (t, *J* = 7.7 Hz, 1H), 7.46 (d, *J* = 7.8 Hz, 1H), 4.25 (d, *J* = 13.3 Hz, 2H), 3.30 (dt, *J* = 3.2, 1.6 Hz, 1H), 2.96 – 2.84 (m, 2H), 1.92 (d, *J* = 12.3 Hz, 2H), 1.75 – 1.63 (m, 2H), 1.48 (s, *J* = 8.1 Hz, 9H); MS (ESI) for [M + H]^+^ (C_24_H_27_N_6_O_3_^+^): calcd. *m*/*z* 447.51; found *m*/*z* 447.50; LC-MS: 95% purity.

To a solution of *tert*-butyl 4-(3-(5-(1*H*-tetrazol-5-yl)­benzo­[*c*]­isoxazol-3-yl)­phenoxy)­piperidine-1-carboxylate (310 mg, 0.671 mmol) in MeOH (3.4 mL) was added a 4 N HCl solution in 1,4-dioxane (1.68 mL, 6.71 mmol). The reaction mixture was stirred at rt for 3 h and concentrated under reduced pressure. The residue was sonicated in a small amount of a mixture of MeOH/H_2_O (1:1) and collected by centrifugation to afford 3-(3-(piperidin-4-yl)­phenyl)-5-(1*H*-tetrazol-5-yl)­benzo­[*c*]­isoxazole hydrochloride (229 mg, 0.574 mmol, 86%) as a yellow solid. ^1^H NMR (400 MHz, DMSO-*d*_6_) δ 8.44 (s, 1H), 8.18 (d, *J* = 8.9 Hz, 1H), 7.96 (d, *J* = 8.2 Hz, 1H), 7.90 (s, 1H), 7.73–7.62 (m, 2H), 7.46 (d, *J* = 7.8 Hz, 1H), 4.14–4.01 (m, 1H), 3.17–2.98 (m, 4H), 2.06 (d, *J* = 13.5 Hz, 2H), 1.96–1.80 (m, 2H); MS (ESI) for [M + H]^+^ (C_19_H_19_N_6_O^+^): calcd. *m*/*z* 347.40; found *m*/*z* 347.40; LC-MS: 96% purity.

To a solution of 3-(3-(piperidin-4-yl)­phenyl)-5-(1*H*-tetrazol-5-yl)­benzo­[*c*]­isoxazole hydrochloride (30.0 mg, 78.4 μmol), Et_3_N (21.9 μL, 0.157 mmol), and HOAc (6.7 μL, 0.12 mmol) in DMF (0.72 mL) was added cyclobutanone (17.6 μL, 0.240 mmol). The resulting mixture was stirred at rt for 30 min, then was added sodium triacetoxyborohydride (33.2 mg, 0.157 mmol). The reaction mixture was stirred at rt for 18 h. Upon completion, the reaction mixture was purified by reverse ISCO silica gel column (MeCN/H_2_O gradient) to afford **14** (31.6 mg, 67.4 μmol, 92%) as a yellow solid. ^1^H NMR (400 MHz, DMSO-*d*_*6*_) δ 8.86 (s, 1H), 8.14–8.00 (m, 3H), 7.91 (dd, *J* = 9.4, 0.7 Hz, 1H), 7.68 (t, *J* = 7.8 Hz, 1H), 7.52 (d, *J* = 7.8 Hz, 1H), 3.65 (dd, *J* = 16.5, 8.4 Hz, 1H), 3.46 (d, *J* = 12.1 Hz, 2H), 3.12–3.01 (m, 2H), 2.93–2.84 (m, 2H), 2.35–2.17 (m, 4H), 2.16–2.00 (m, 3H), 1.83–1.67 (m, 2H); ^13^C NMR (101 MHz, DMSO-*d*_6_) δ 178.55, 157.47, 146.41, 145.16, 130.62, 130.16, 129.89, 127.65, 125.68, 125.22, 121.57, 121.41, 117.03, 113.99, 58.63, 49.29, 38.91, 29.85, 25.55, 13.59; MS (ESI) for [M + H]^+^ (C_23_H_25_N_6_O^+^): calcd. *m*/*z* 401.49; found *m*/*z* 401.50; LC-MS: 97% purity.

### ^3^H-Inositol Metabolic Labeling and HPLC

Human U251-MG glioblastoma cells (ECACC 09063001) were grown in DMEM supplemented with 10% FBS. The medium was changed to inositol-free DMEM, and cells were inositol-starved for 1–3 days. Cells were maintained in inositol-free DMEM medium, treated with the indicated inhibitor or DMSO for 1–2.5 h, and supplemented with 100 μCi ^3^H-inositol (PerkinElmer) for 48 or 72 h, as indicated on each figure. Cells were then washed in PBS and lysed in 200 μL HCl (0.5 M), 250 μL of MeOH was added to each well of a 6-well plate (approximately 10^6^ cells), followed by 125 μL of 2 M KCl.^46^ Lysates were scraped and sonicated on ice for three 1 min cycles. Inositol phosphate (IP) extracts were cleaned of lipids by adding 250 μL of CHCl_3_, vortexing and centrifuging samples for 2 min at 5000 rpm to separate upper aqueous and lower organic phases, upper phase contains soluble IPs. This extraction was repeated once. Samples were dried in a SpeedVac, resuspended in 10 mM ammonium phosphate (pH= 3.5), and filtered. HPLC separation and detection of the inositol species on the upper phase used a Partisphere SAX 4.6 mm × 250 mm column, eluted with 10 mM (buffer A) to 1.3 M (buffer B) ammonium phosphate (pH = 3.8) and online flow scintillation analyzer (FSA, PerkinElmer). Elution of [^32^P]-labeled IP_4_, IP_5_, and IP_6_ standards confirmed the identity of each peak off the HPLC. Quantification represents three biologically independent metabolic labeling samples with error representing the standard deviation of the mean.

### RNA-Seq and Gene Set Enrichment Analysis

DNase-treated RNA prepared from one well of a six-well plate (approximately 10^6^ cells) was extracted using the Quick-RNA MiniPrep kit (Zymo Research) from human U251-MG glioblastoma cells (ECACC 09063001) after 48 h of treatment with compound **1**. PolyA-selected RNA-seq libraries were prepared and sequenced at the Vanderbilt Technologies for Advanced Genomics (VANTAGE) core on an Illumina NextSeq500. Bases and reads with low-quality scores or reads, were removed, and Illumina library adapter sequences were trimmed using bbDuk (https://jgi.doe.gov/data-and-tools/software-tools/bbtools/bb-tools-user-guide/bbduk-guide/). The resulting files were mapped to hg38 using HiSat2,^47^ and poorly mapped reads were removed with SamTools.^48^ Reads mapping to RefSeq genes were counted using HTSeq-count^49^ and differential expression was assessed using DESeq2 ^50^ with Apeglm LFC Shrinkage,^51^ expressed as compound **1**/DMSO in all log 2 fold-change expressions. Gene sets from the Molecular Signatures Database (version 6.1) were used for Gene Set Enrichment Analysis (GSEA). These data are provided as supplemental spreadsheets, and all original FASTQ files and underlying count data are available upon request.

### IPMK IC_50_ Value Determinations

IPMK IC_50_ values were determined using the purified recombinant human IPMK kinase domain expressed in in the Kinase-Glo assay, as previously described.^2,52,53^ This human IPMK kinase domain (130 nM; however, a fraction of this preparation is inactive enzyme^53^) was used in reaction buffer (20 mM HEPES, pH 6.8; 100 mM NaCl; 6.0 mM MgCl_2_; 20 μg/mL BSA; 1.0 mM DTT) containing 12.5 μM Ins­(1,4,5)­P_3_ kinase substrate and 10 μM ATP at room temperature, and the reactions were analyzed after 12 min. These assay conditions were optimized with respect to reaction time, IPMK enzyme concentration, and IP_3_ concentration to determine the IC_50_ values for these ATP-competitive compounds.

### IP6K2 IC_50_ Value Determinations

IP6K2 IC_50_ values were determined using the purified recombinant human IP6K2 kinase domain expressed in^54^ in a 384-well clear polystyrene plate (VWR 82051-264). This human IP6K2 kinase domain (100 nM) was used at 37 °C in 50 μL reaction mixtures containing 5.0 μM human Dipp1, 50 mM Tris (pH 6.9), 10 mM MgCl_2_, 0.02% Triton X-100, 25 μM InsP6, and 50 μM ATP for 30 min. Pi release was determined with a malachite green colorimetric assay. Reactions were quenched by the addition of 12.5 μL of phosphate detection reagent (MAK3071KT, Sigma-Aldrich). Pi release was quantified from the absorbance at 620 nm by using appropriate standards.

### Pharmacokinetic Studies

All the procedures related to animal handling, care, and treatment in this study were performed according to guidelines approved by the Institutional Animal Care and Use Committee (IACUC) of Pharmaron (PK-R-06012023 and PK-M-07182023), following the guidance of the Association for Assessment and Accreditation of Laboratory Animal Care (AAALAC). A group of 3 male CD1 mice (2 for exploratory PK) were dosed intravenously (IV) or intraperitoneally (IP) with a solution formulation of the tested compounds in 10% NMP, 5% Solutol in normal saline (v/v/v, 10:5:85). From each mouse, blood samples (30 mL/sample) were collected from the orbital vein such that samples were obtained at 0.083, 0.25, 0.5, 1, 2, 4, 8, and 24 h post dose. At each time point, blood samples were collected from three mice in labeled microcentrifuge tubes containing EDTA-K2 as an anticoagulant. Plasma samples were separated by centrifugation of whole blood and stored at −75 ± 15 °C until bioanalysis. All samples were processed for analysis by precipitation using acetonitrile (ACN) and analyzed with a fit-for-purpose LC/MS/MS method (the below quantifiable limit (BLOQ) was 5.0 ng/mL). Pharmacokinetic parameters were calculated by a noncompartmental analysis model using WinNonlin 8.3.

### Protein Kinase Profiling

Protein kinase profiling of compound **14** was executed by Carna Biosciences. Briefly, compound **14** was prepared by dissolving it in dimethyl sulfoxide (DMSO) and then further diluting it in DMSO to a concentration of 25 μM. That stock solution was further diluted 25-fold with kinase assay buffer (20 mM HEPES, 0.01% Triton X-100, 2 mM DTT, pH 7.5) to make the final 1 μM compound **14** solution. Reference compounds for the assay controls were prepared similarly. The off-chip Mobility Shift Assay (MSA) was prepared with 5 μL of 4× compound solution, 5 μL of 4× substrate/ATP/metal solution, and 10 μL of 2× kinase solution, prepared with assay buffer (20 mM HEPES, 0.01% Triton X-100, 2 mM DTT, pH 7.5). The components were mixed and incubated in a polypropylene 384-well microplate for 1 h or 5 h at room temperature, depending on the kinase being tested. 60 μL of termination buffer (QuickScout Screening Assist MSA; Carna Biosciences) was added to the well to stop the reactions, and the mixture was applied to a LabChip3000 system (Caliper Life Sciences. The product and substrate peptide peaks were separated and quantitated. The kinase reactions were evaluated by the product ratio, calculated from the peak heights of the product (P) and substrate (S) peptides (P/(P + S)). Product ratio values for the control reaction were set as 0% inhibition, and the readout value of the background (no kinase added) was set as 100% inhibition. The percent inhibition of each test solution was then calculated as reported in Figure 8A. The phylogenetic tree was generated using KinMap at https://bmcbioinformatics.biomedcentral.com/articles/10.1186/s12859-016-1433-7.

### Western Blots

About 3.5 × 10^6^ U251 cells were seeded in 15 cm plates containing 30 mL of DMEM supplemented with 4.5 g/L d-glucose, l-glutamine, 10% FBS, 1% nonessential amino acids, and 1% penicillin–streptomycin (all from Thermo Fisher). Cells were treated with 10 μM, 30 μM, or 50 μM of compound **14** or DMSO for 24 h, and then the cells were washed with PBS and lysed using RIPA buffer. Protein concentration was equalized using a Bradford assay, and samples were run on SDS-PAGE at 150 V for 1 h. Protein transfer was performed at 20 V for 1 h, membranes were blocked with 7 mL of EveryBlot blocking buffer (Bio-Rad) for 10 min. Primary antibodies used were AKT (#9272), Actin (#3700), and phospho-AKT (Ser473, #4060) at a 1:1000 dilution in EveryBlot blocking buffer, and phospho-AKT (Thr308, #13038) at a 1:400 dilution; all antibodies were from Cell Signaling Technology. After overnight incubation at 4 °C, membranes were washed and incubated with secondary antibodies (1:10,000 dilution for AKT, Actin, and P-AKT Ser473; 1:4000 for P-AKT Thr308) for 1 h. Detection was performed using Clarity Western ECL substrate (Bio-Rad) according to the manufacturer’s instructions and imaged using the Bio-Rad ChemiDoc system.

### Reverse-Transcriptase Semiquantitative Polymerase Chain Reaction (RT-qPCR)

About 2 million human U251MG cells were treated as indicated for 24 h, and total RNA was purified (RNeasy, Qiagen). First-strand cDNA was synthesized from 2 μg of total RNA using random-primed reverse transcription (Verso cDNA Synthesis, Thermo Scientific) using a thermal cycler according to manufacturer’s instructions. First-strand cDNA synthesis was done at 42 °C for 30 min, followed by 2 min of inactivation at 95 °C. Triplicate biological replicates with 2 technical replicates were used for each RT-qPCR condition; no data points were excluded from the analysis. *GAPDH* was the reference gene for normalization, and all primers were validated at serially diluted cDNA concentrations to produce a single PCR product in the dissociation curve. Primer sequences (all human genes) were: Cyclophilin (*PPIA*) Forward: 5′-GCGACTTTCTGGAGTTTATTTCA-3′; Cyclophilin (*PPIA*) Reverse: 5′-TTTCATTGCTTCTGGGTTCC-3′ *GAPDH* Forward: 5′-CAAGGTCATCCATGACAACTTTG-3′; *GAPDH* Reverse: 5′-GGCCATCCACAGTCTTCTGG-3′; *SLCO1B1* Forward: 5′-CGTAGAGCAACAGTATGGTCAGC-3′; *SLCO1B1* Reverse: 5′-TTGGCAATTCCAACGGTGTTCAG-3′; *NR0B2* Forward: 5′-GCTTAGCCCCAAGGAATATGC-3′; *NR0B2* Reverse: 5′-TTGGAGGCCTGGCACATC-3′; *CYP11A1* Forward: 5′-GGGTCGCCTATCACCAGTATT-3′; *CYP11A1* Reverse: 5′-GCTGCCGACTTCTTCAACAG-3′; *StAR* Forward: 5′-CCACAAACGGCCAAGCA-3′; *StAR* Reverse: 5′-CGCCATCACTCACTGTGCAA-3′. A CFX96 Real-Time qPCR instrument (Bio-Rad, Hercules, CA) was used for real-time data acquisition using the following cycling parameters: predenaturation 95 °C for 5 min, denaturation 95 °C for 10 s, annealing and extension 60 °C for 30 s for a total of 40 cycles. A melt curve step at 65 °C for 5 s and 56 °C for 50 s in every run further validated the specificity of primers to produce only one PCR product. The ΔΔCt method using a reference gene (*Cyclophilin, PPIA*, or *GAPDH*) Ct subtracted from the Ct of the target gene of interest normalized transcript levels for each cDNA sample. The data were analyzed in GraphPad Prism by one-way ANOVA with Dunnett’s correction for multiple comparisons from all triplicate biological and technical duplicates. All raw RT-qPCR data are available on request.

### Graphical Software

Graphs were prepared using SigmaPlot v14 and GraphPad Prism 9; IC_50_ data were calculated using GraphPad Prism 9.

## Supplementary Material





## Notes

Gene set enrichment bar code plots for **1** (Figure S1), IP6K2 IC_50_ data for compounds **1**–**4**, **6**–**14** (Table S1), NMR spectra and analytical HPLC spectra for compounds **11**–**14** (Figures S2–S13), Western analysis of P-AKT (Figure S14), RT-qPCR analysis of *SF-1* target genes (Figure S15) (PDF)
Molecular formula strings (CSV)

## □

Onkure Therapeutics 6707 Winchester Circle, Boulder, Colorado 80301, United States

## ■

University of Pittsburgh, Pittsburgh, Pennsylvania 15260, United States

## Abbreviations

AGC: containing PKA, PKG, and PKC families
CAMK: calcium/calmodulin-dependent protein kinase
CK1: casein kinase 1
CMGC: containing CDK, MAPK, GSK3, and CLK families
DIAD: diisopropyl azodicarboxylate
EMT: epithelial-to-mesenchyme transition
GSEA: gene set enrichment analysis
InsP_3_ or Ins­(1,4,5)­P_3_: inositol 1,4,5-trisphosphate
InsP_4_: inositol tetraphosphate
InsP_5_: inositol pentakisphosphate
InsP_6_: inositol hexakisphosphate
IP6K: inositol hexakisphosphate kinase
IPMK: inositol phosphate multikinase
IV: intravenous
MeCN: acetonitrile
PPh3: triphenylphosphine
*i*-PrOH: isopropyl alcohol
TK: tyrosine kinase
TKL: tyrosine kinase-like
