# Supplementary material for: Design, Synthesis, and Cellular Characterization of a New Class of IPMK Kinase Inhibitors
Source: J Med Chem. 2025 Jul 25;68(15):15446–60. doi: 10.1021/acs.jmedchem.5c00015 (PMC12362615; doi:10.1021/acs.jmedchem.5c00015)
Supplement: Supplementary file 1 [file jm5c00015_si_001.pdf]

Supplemental Information

Design, synthesis and cellular characterization of a new class of IPMK kinase inhibitors

Yubai Zhou[1#], Pratima Chapagain[2,3#], Desmarini Desmarini[4,5], Dilipkumar Uredi[1], Michael A. Stashko[1], Hundaol Huluka[2,3], Lucia E. Rameh[2,6], Julianne T. Djordjevic[4,5,7], Raymond D. Blind[2,3*] and Xiaodong Wang[1,8*]

[1]Center for Integrative Chemical Biology and Drug Discovery, Division of Chemical Biology and Medicinal Chemistry, Eshelman School of Pharmacy, University of North Carolina at Chapel Hill, Chapel Hill, NC 27599, USA.

[2]Department of Medicine, Division of Diabetes, Endocrinology & Metabolism, Vanderbilt University Medical Center, Nashville, TN 37232, USA.

[3]Departments of Biochemistry & Pharmacology, Vanderbilt University School of Medicine, Nashville, TN 37232, USA.

[4]Centre for Infectious Diseases and Microbiology, The Westmead Institute for Medical Research, Westmead, NSW 2145, Australia.

[5]Sydney Institute for Infectious Diseases, Faculty of Medicine and Health, University of Sydney, Sydney, NSW 2006, Australia.

[6]Department of Biochemistry & Molecular Biology, University of South Alabama, Mobile, AL 36688

[7] Western Sydney Local Health District, Westmead Hospital, Westmead, NSW 2145, Australia

[8]Lineberger Comprehensive Cancer Center, University of North Carolina at Chapel Hill, Chapel Hill, NC 27599, USA.

#Contributed equally

*Correspondence:

xiaodonw@unc.edu

ray.blind@vanderbilt.edu

Supplemental Figure Index

Fig S1; Gene Set Enrichment bar code plots for **1** Page S2

Table S1; IP6K2 IC_{50} data Page S3

Fig S2-S13; ^{1}H, ^{13}C NMR and HPLC Spectra Pages S4-S11

Fig S14; Western analysis of P-AKT Page S12

Fig S15; RT-qPCR analysis of SF-1 target genes Page S13

Supplemental Figure S1:

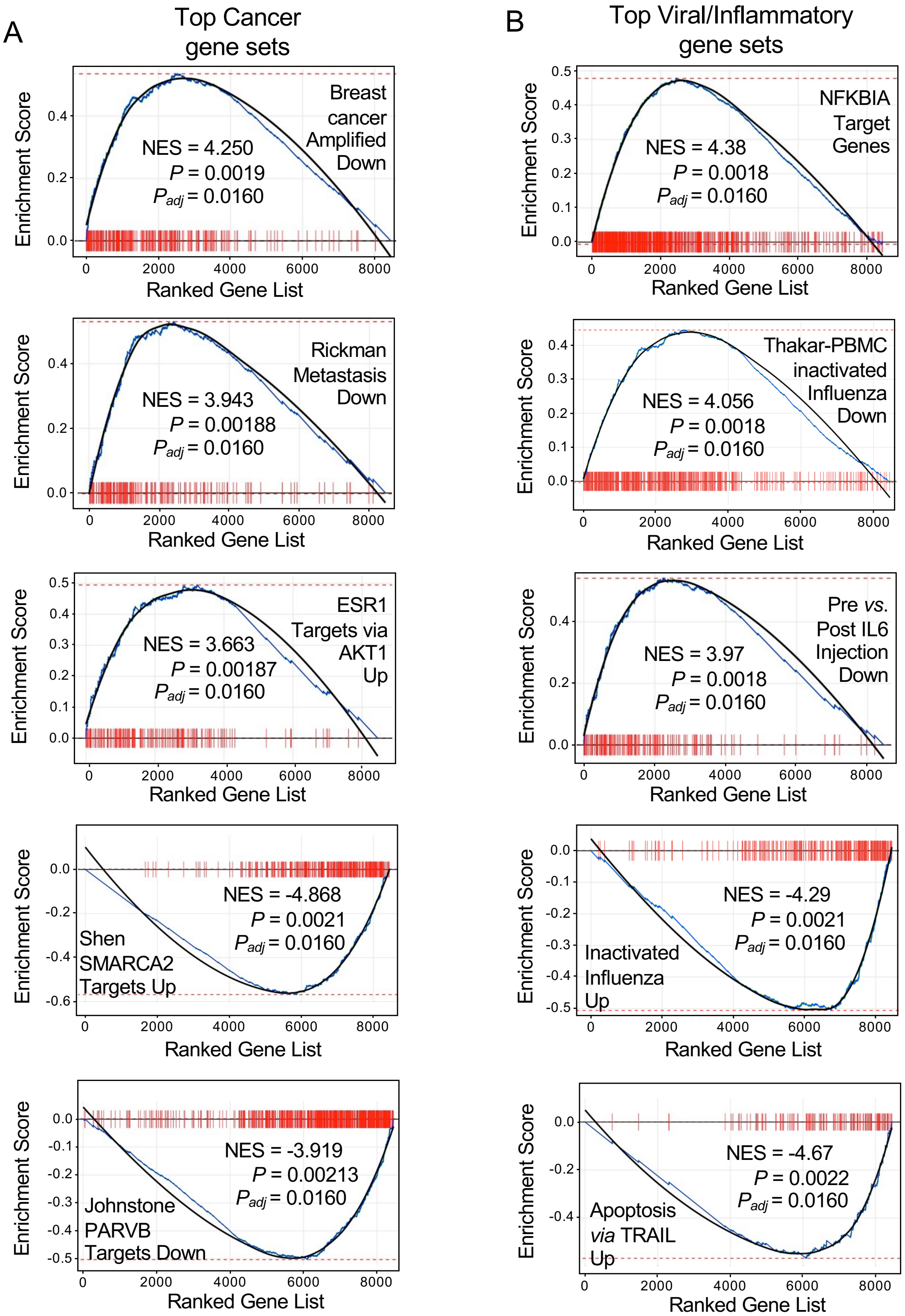

Supplemental Figure S1. *Gene sets enriched in Compound 1-regulated transcripts*. Bar code GSEA plots from **A**. the top cancer-related gene sets and **B**. the top viral/inflammatory gene sets from the Molecular Signatures database. Normalized enrichment scores (NES) and *p*-values (both unadjusted and adjusted) are indicated in each enrichment plot.

Table S1. IP6K2 IC_{50} values.

Compound	IP6K2 IC50 (nM)[a]	IPMK IC50 (nM)[a]	IP6K2/IPMK
**1**	248±119	26.2±1.2	9.5
**2**	265±20.0	166±19.6	1.6
**3**	73.0±27.4	215±37.5	0.3
**4**	113±69.5	24.7±1.6	4.6
**6**	125±66.2	36.0±5.5	3.5
**7**	146±97.1	18.6±1.4	7.8
**8**	91.7±50.9	18.3±1.6	5.0
**9**	942±348	185±15.5	5.1
**10**	297±141	58.2±3.4	5.1
**11**	107±64.4	30.2±1.7	3.5
**12**	153±79.8	31.3±4.0	4.9
**13**	129±44.1	72.1±5.2	1.8
**14**	374±159	31.6±2.4	12

[a] Values are the mean of three or more independent assays.

Supplemental Figure S2:

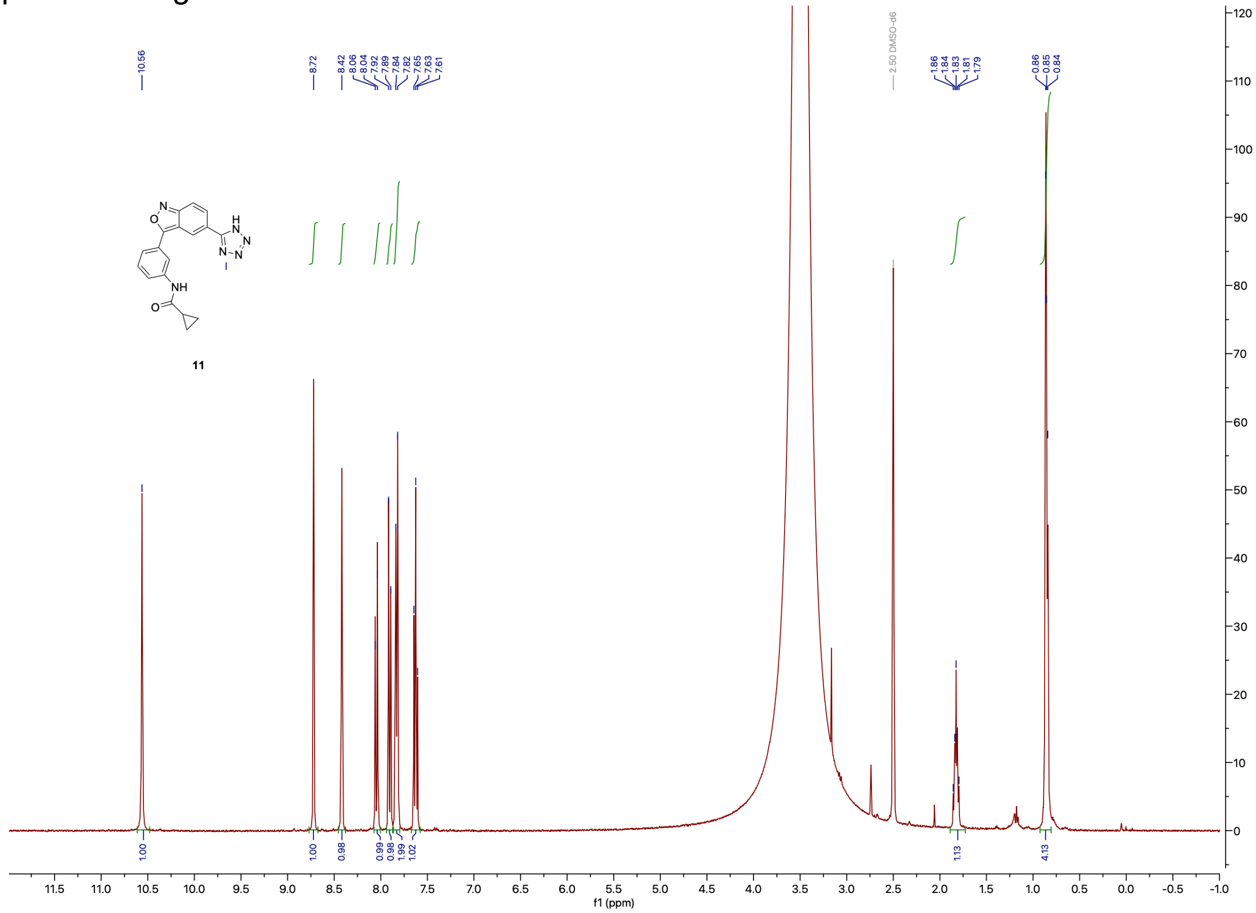

Supplemental Figure S3:

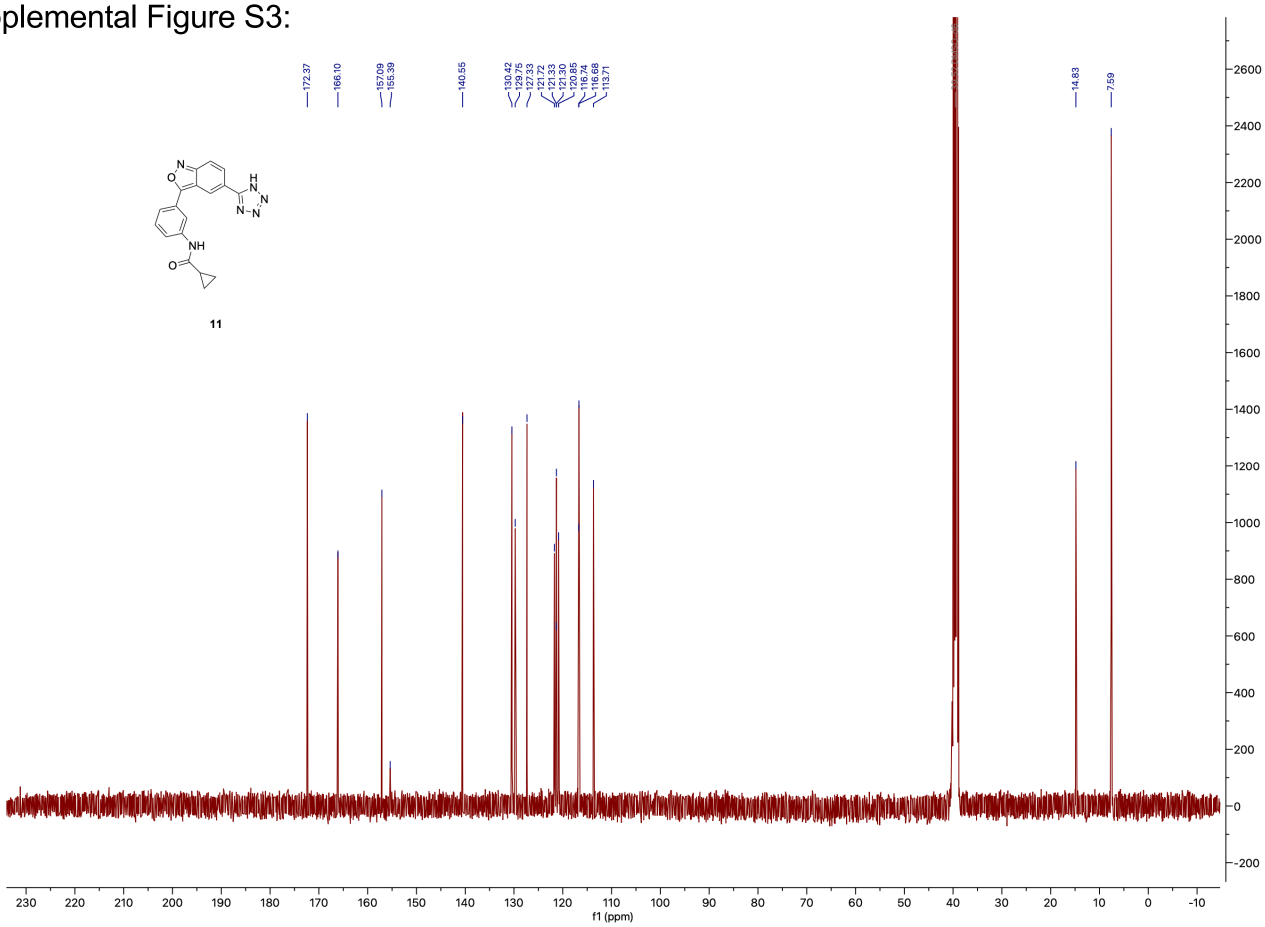

Supplemental Figure S4:

Single Injection Report

**Data file:**	C:\CDSProjects\1260 project\Results\Megan\20220801_purity\20220801_purity.rslt\yz-12.dx		
**Sample name:**	yz-12		
**Description:**			
**Sample amount:**	0.000	**Sample type:**	Sample
**Instrument:**	1260	**Location:**	P1-A2
**Injection date:**	2022-08-01 14:38:51-04:00	**Injection:**	1 of 1
**Acq. method:**	1260_ShortPurity_1_214.amx	**Injection volume:**	5.000
**Analysis method:**	1260_ShortPurity_LC AreaPercent_DefaultMethod.pmx	**Acq. operator:**	SYSTEM
**Last changed:**	2019-09-11 09:03:16-04:00		

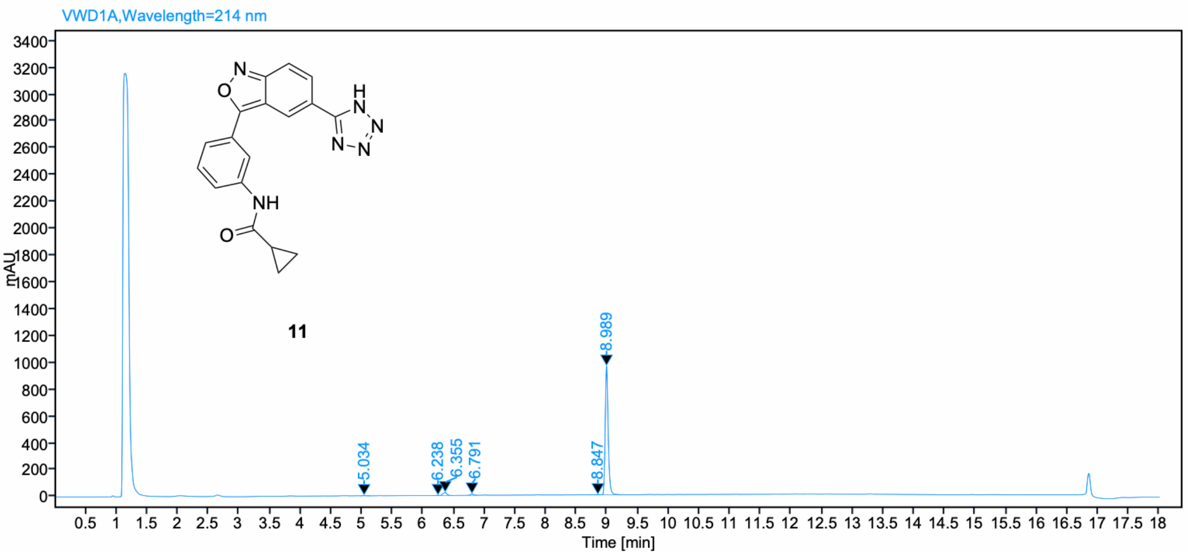

Signal: VWD1A,Wavelength=214 nm

RT [min]	Type	Width [min]	Area	Height	Area%	Name
5.034	VB	0.1648	10.7755	3.1149	0.3195	
6.238	BV	0.1353	5.7741	1.7833	0.1712	
6.355	VB	0.2264	99.9035	23.1585	2.9626	
6.791	BB	0.1604	33.7091	11.2338	0.9996	
8.847	BV	0.1957	10.3484	2.2347	0.3069	
8.989	VV	0.3186	3211.6748	960.9215	95.2402	
		Sum	3372.1853			

Supplemental Figure S5:

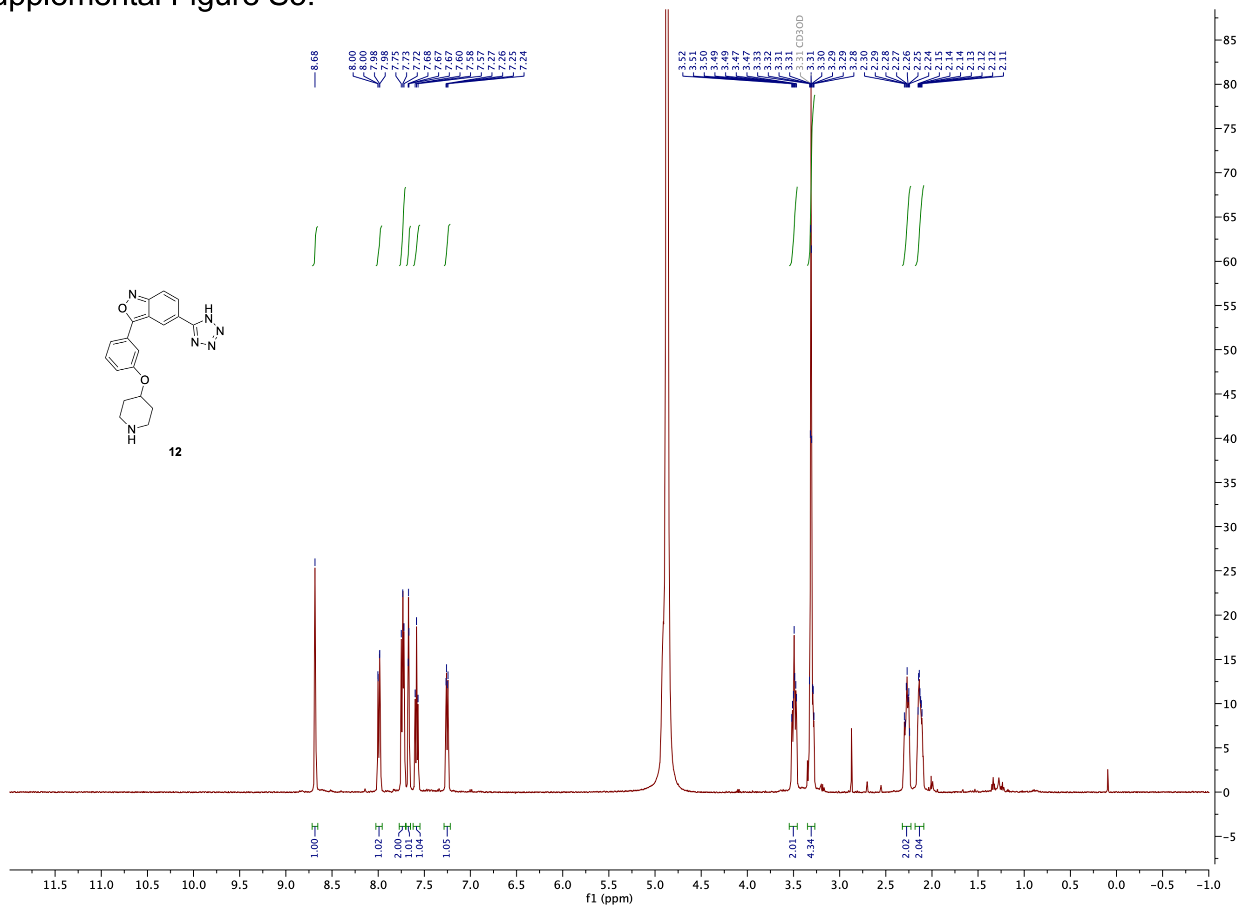

Supplemental Figure S6:

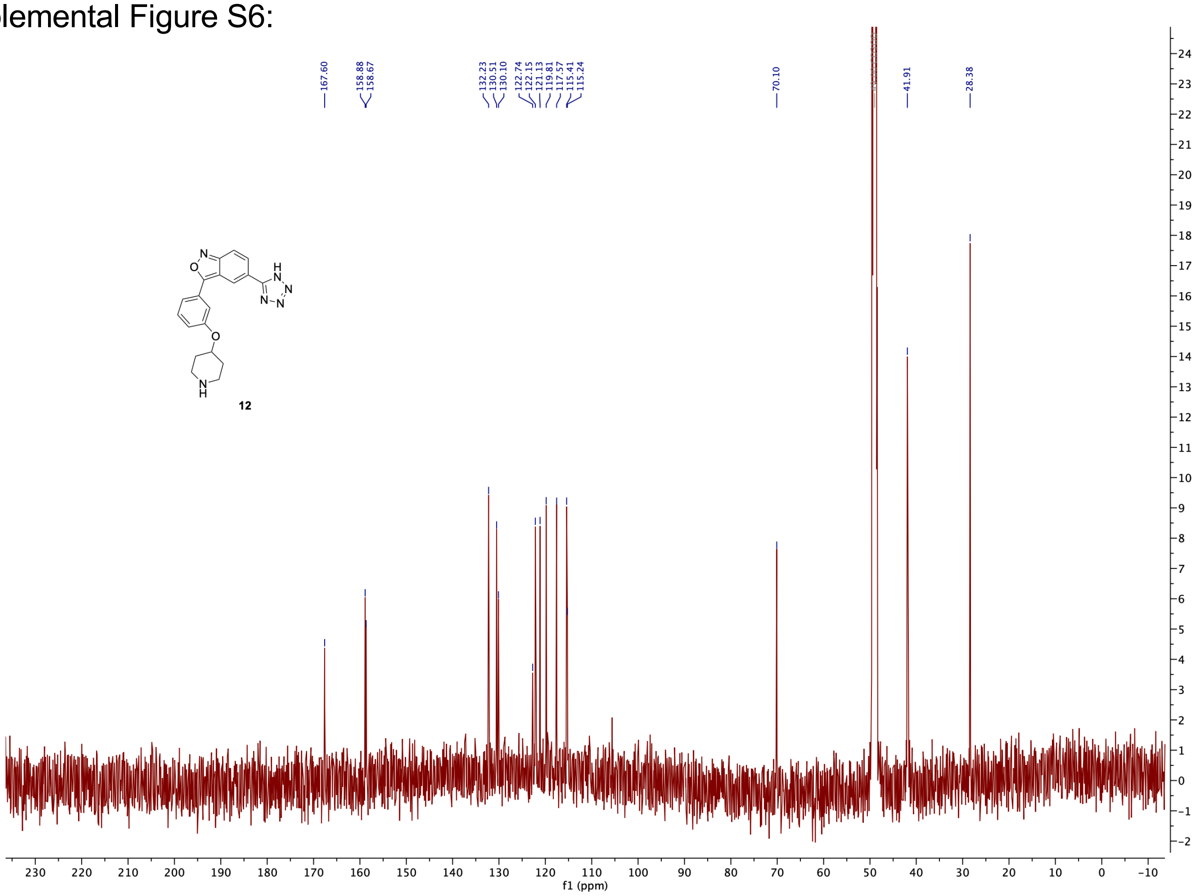

Supplemental Figure S7:

Single Injection Report

Data file: C:\CDSProjects\1260 project\Results\Megan\20220801_purity\20220801_purity.rslt\yz-14.dx

Sample name: yz-14

Description:

Sample amount: 0.000 **Sample type:** Sample

Instrument: 1260 **Location:** P1-A3

Injection date: 2022-08-01 14:57:35-04:00 **Injection:** 1 of 1

Acq. method: 1260_ShortPurity_1_214.amx **Injection volume:** 5.000

Analysis method: 1260_ShortPurity_LC AreaPercent_DefaultMethod.pmx **Acq. operator:** SYSTEM

Last changed: 2019-09-11 09:03:16-04:00

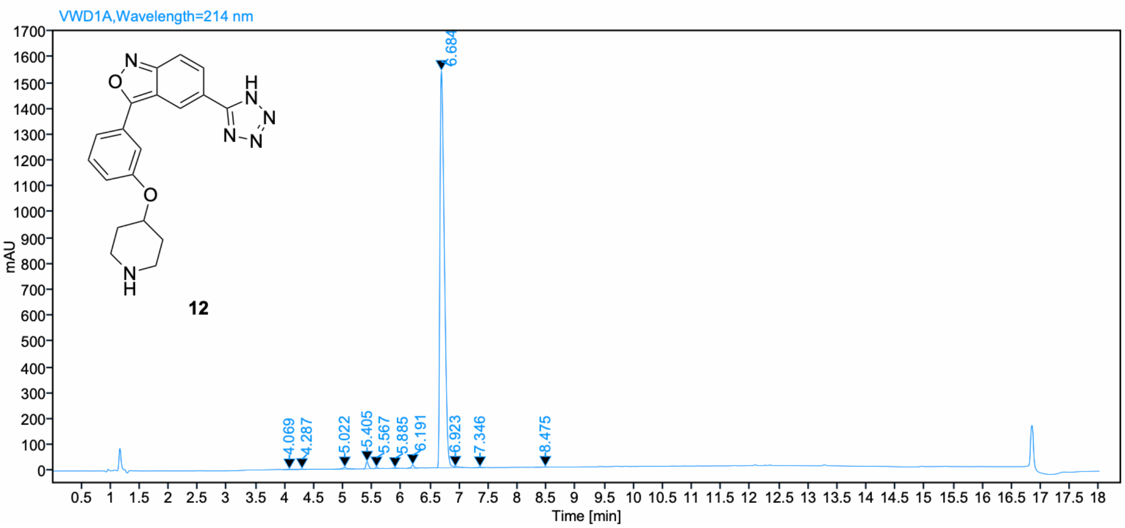

Signal: VWD1A,Wavelength=214 nm

RT [min]	Type	Width [min]	Area	Height	Area%	Name
4.069	VV	0.2060	16.5563	1.8763	0.1980	
4.287	VV	0.1976	13.1358	2.4393	0.1571	
5.022	BV	0.3709	34.1304	6.8467	0.4081	
5.405	BV	0.1797	87.4294	27.9952	1.0455	
5.567	VV	0.1222	18.2213	5.5168	0.2179	
5.885	VB	0.1169	6.7864	1.8482	0.0812	
6.191	BV	0.1909	44.1208	13.0039	0.5276	
6.684	BV	0.3199	8106.3701	1535.0855	96.9354	
6.923	VB	0.1578	20.3746	3.6141	0.2436	
7.346	BV	0.2147	8.3466	2.0757	0.0998	
8.475	BV	0.2343	7.1801	1.9137	0.0859	
		Sum	8362.6519			

Supplemental Figure S8:

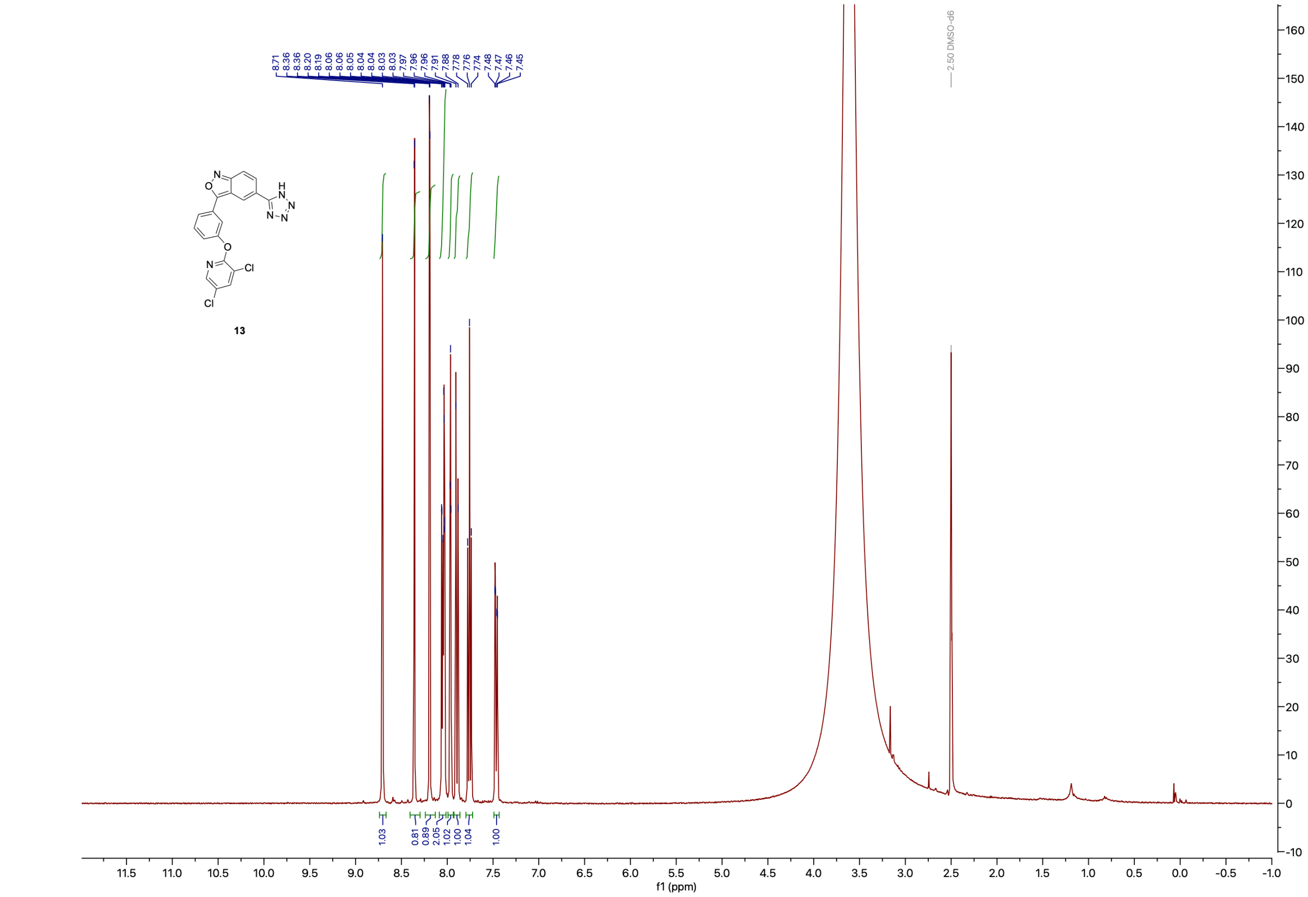

Supplemental Figure S9:

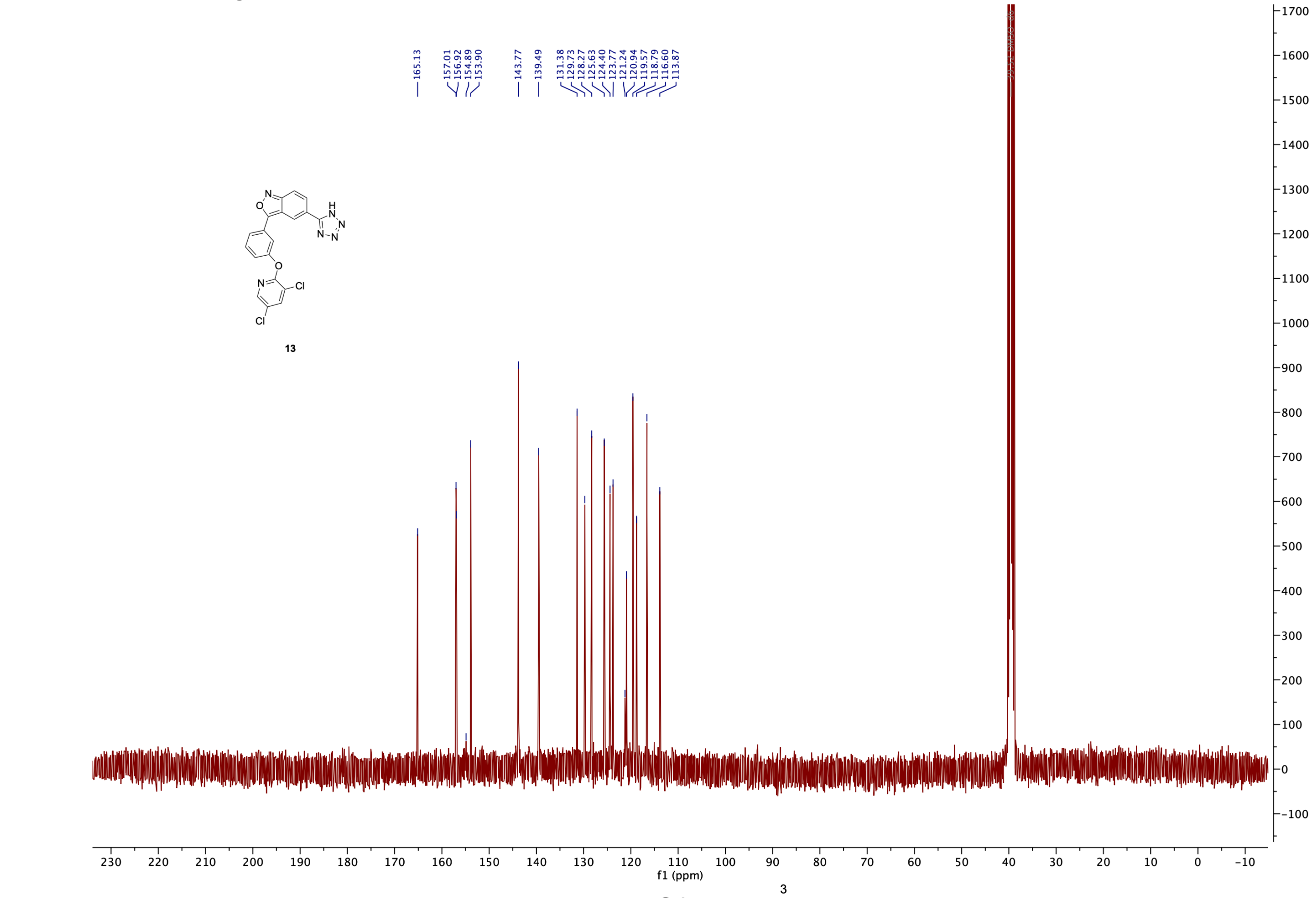

Supplemental Figure S10:

Single Injection Report

**Data file:**	C:\CDSProjects\1260 project\Results\Yubai\20220728_purity\20220728_purity.rslt\yz-15.dx		
**Sample name:**	yz-15		
**Description:**			
**Sample amount:**	0.000	**Sample type:**	Sample
**Instrument:**	1260	**Location:**	P1-A5
**Injection date:**	2022-07-28 16:47:13-04:00	**Injection:**	1 of 1
**Acq. method:**	1260_ShortPurity_1_214.amx	**Injection volume:**	5.000
**Analysis method:**	1260_ShortPurity_LC AreaPercent_DefaultMethod.pmx	**Acq. operator:**	SYSTEM
**Last changed:**	2019-09-11 09:03:16-04:00		

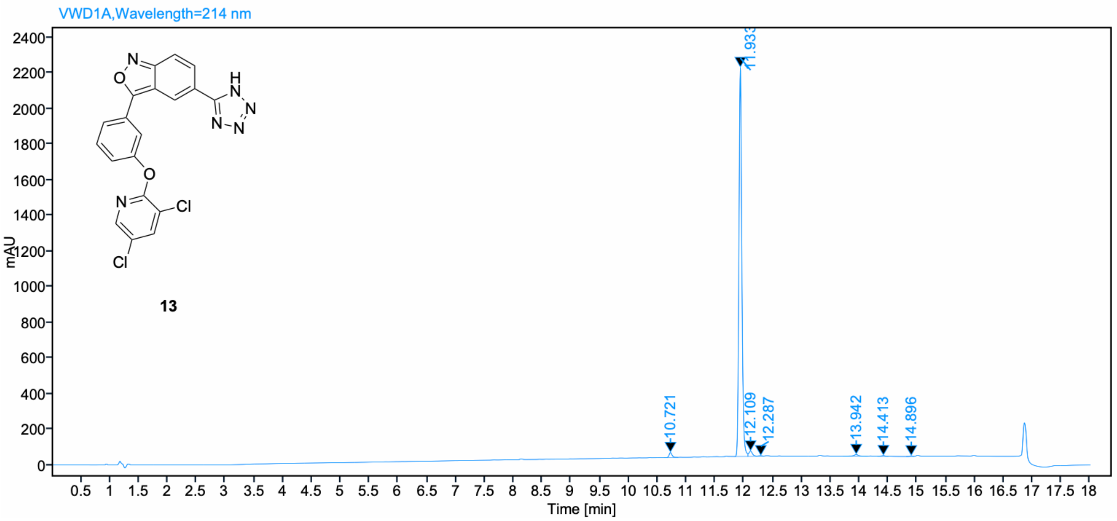

Signal: VWD1A,Wavelength=214 nm

RT [min]	Type	Width [min]	Area	Height	Area%	Name
10.721	BV	0.2440	100.2968	27.9391	1.2538	
11.933	BV	0.2303	7662.8868	2183.3240	95.7894	
12.109	VV	0.1526	129.7011	29.7469	1.6213	
12.287	VV	0.1194	16.1361	2.9984	0.2017	
13.942	VV	0.4285	57.2238	9.3064	0.7153	
14.413	VV	0.1850	22.4423	4.2305	0.2805	
14.896	BV	0.1376	11.0331	3.3283	0.1379	
		Sum	7999.7200			

Supplemental Figure S11:

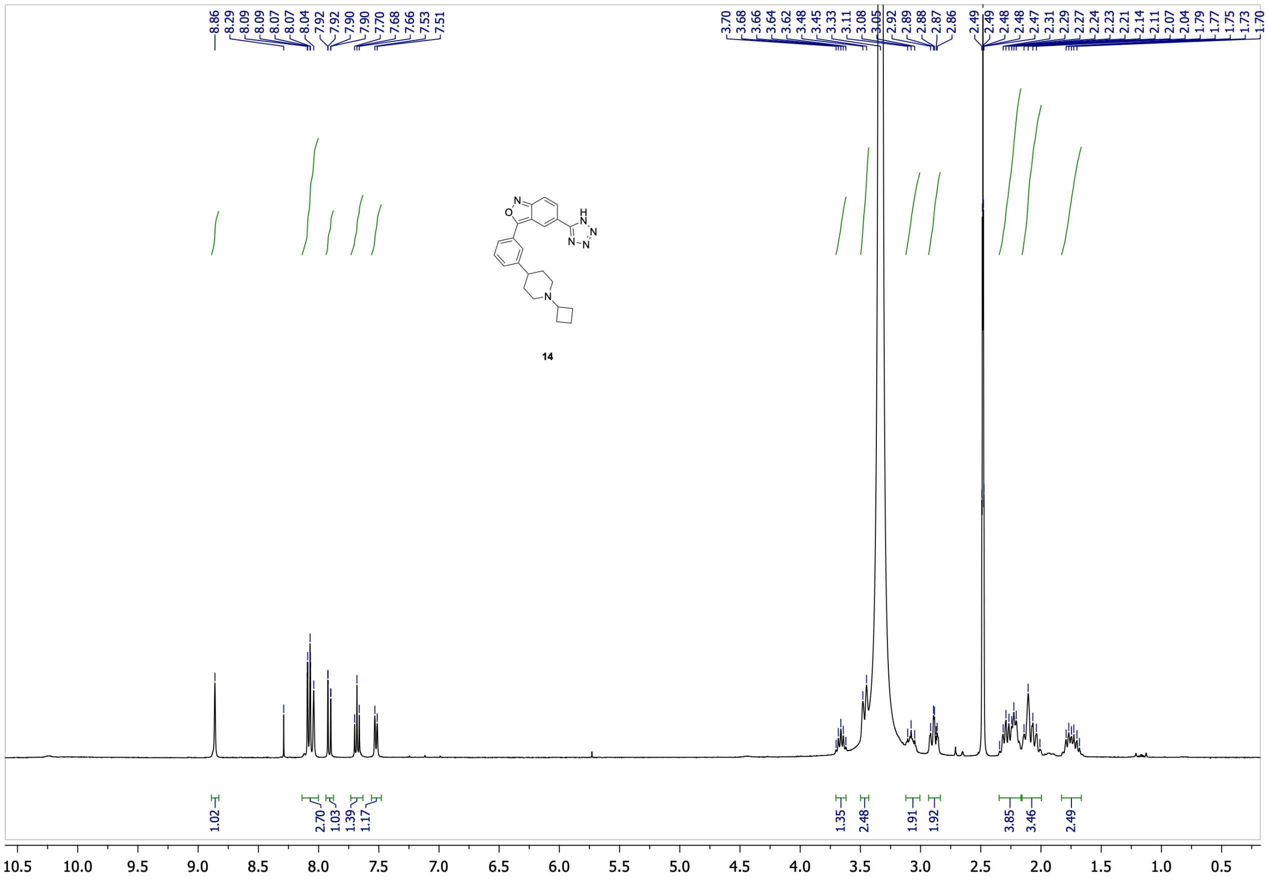

Supplemental Figure S12:

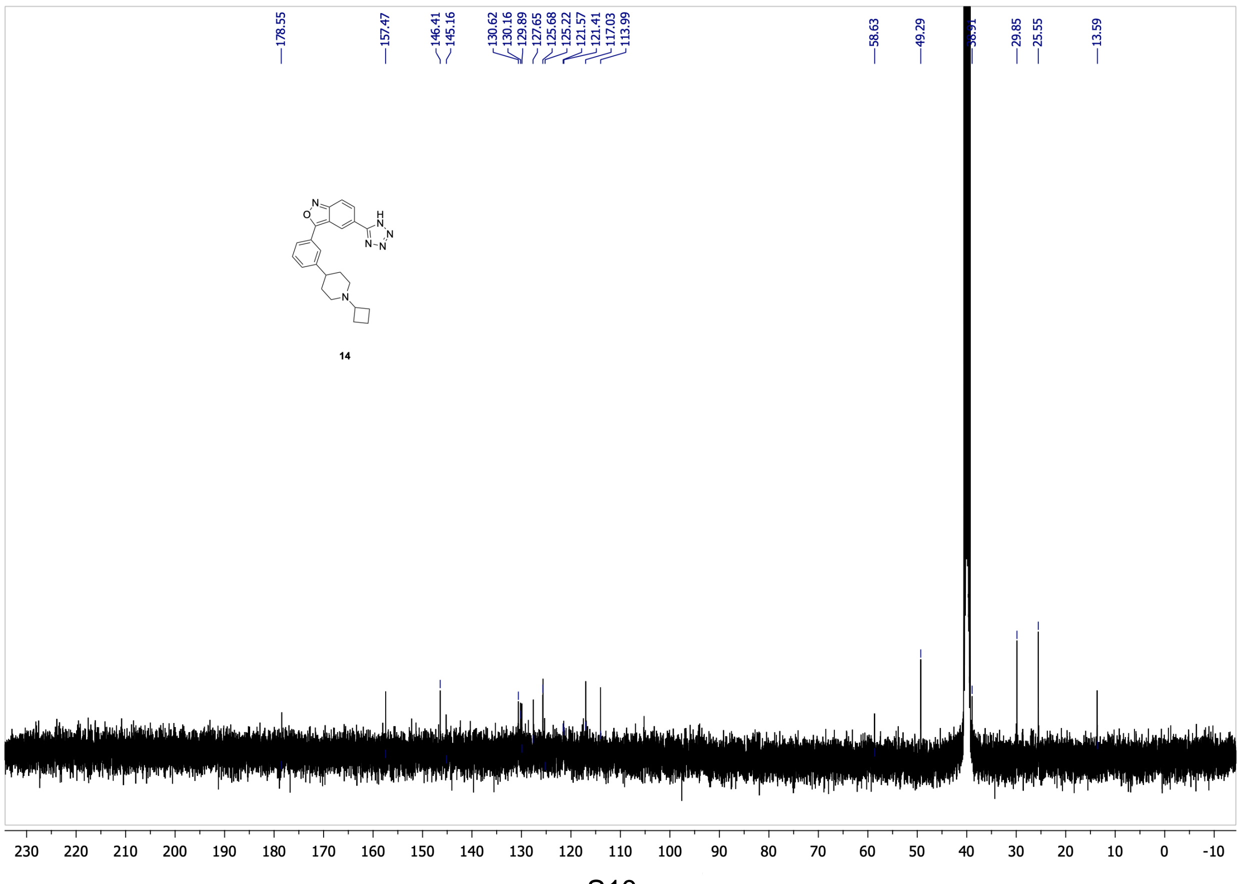

Supplemental Figure S13:

Single Injection Report

**Data file:**	C:\CDSProjects\1260 project\Results\Yubai\20220728_purity\20220728_purity.rslt\yz-16.dx		
**Sample name:**	yz-16		
**Description:**			
**Sample amount:**	0.000	**Sample type:**	Sample
**Instrument:**	1260	**Location:**	P1-A6
**Injection date:**	2022-07-28 17:05:54-04:00	**Injection:**	1 of 1
**Acq. method:**	1260_ShortPurity_1_214.amx	**Injection volume:**	5.000
**Analysis method:**	1260_ShortPurity_LC AreaPercent_DefaultMethod.pmx	**Acq. operator:**	SYSTEM
**Last changed:**	2019-09-11 09:03:16-04:00		

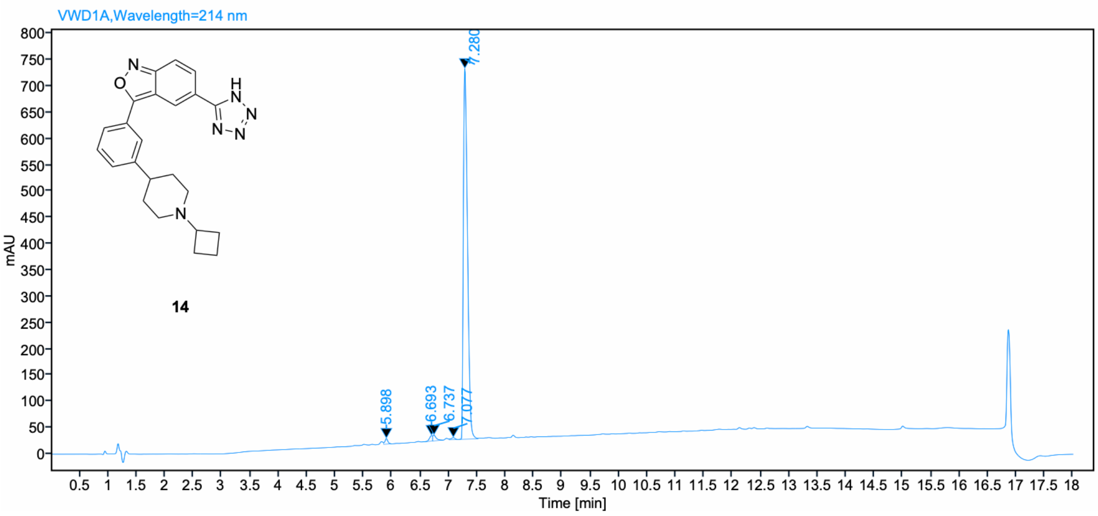

Signal: VWD1A,Wavelength=214 nm

RT [min]	Type	Width [min]	Area	Height	Area%	Name
5.898	VB	0.1447	37.5738	10.6581	1.0153	
6.693	BV	0.1802	38.0469	10.6442	1.0281	
6.737	VB	0.1812	45.8488	11.0247	1.2389	
7.077	VB	0.1595	18.5758	3.8534	0.5019	
7.280	BV	0.3431	3560.7368	704.8773	96.2158	
		Sum	3700.7821			

Supplemental Figure S14:

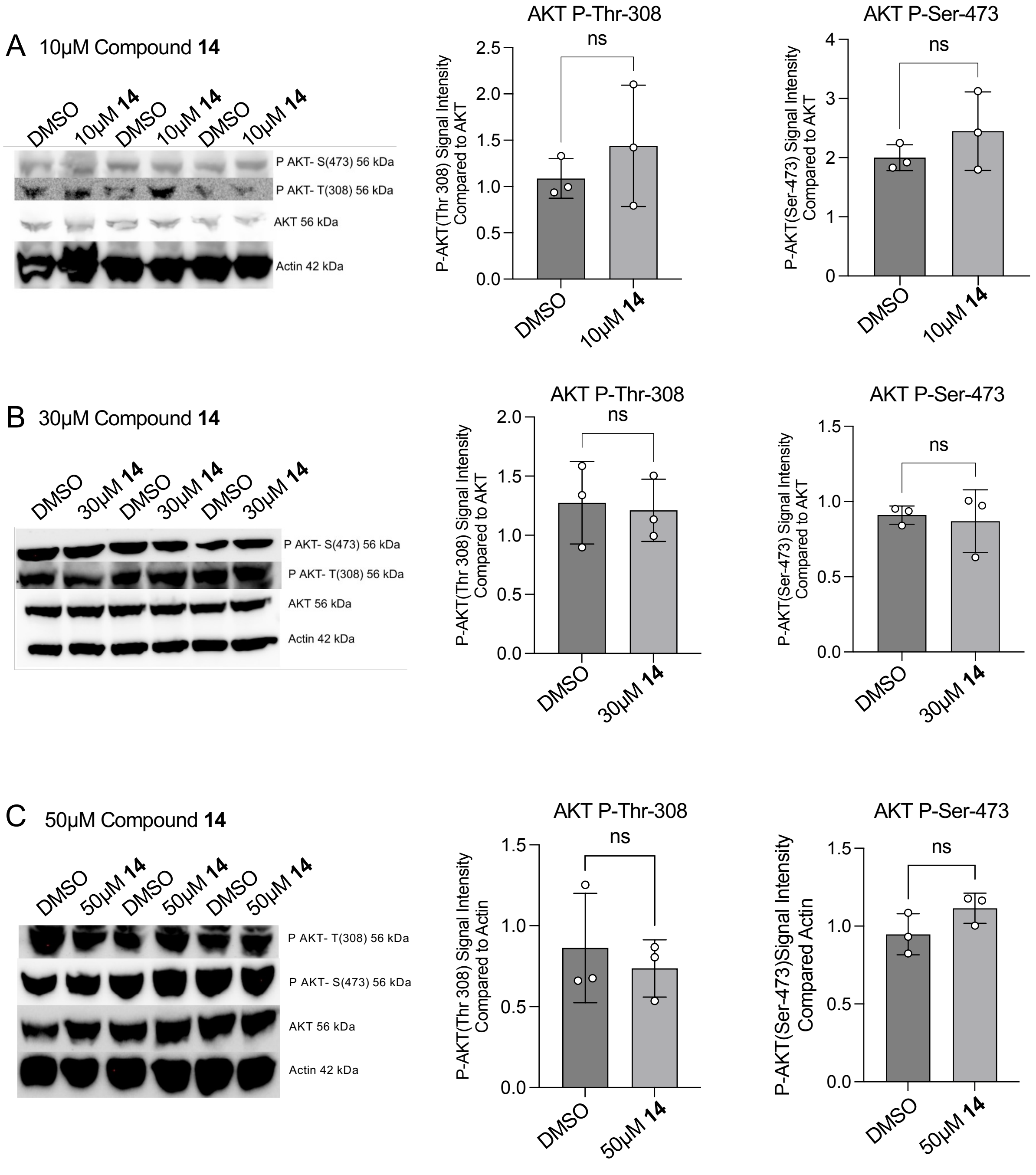

Supplemental Figure S14. *Compound 14 does not affect apparent AKT phosphorylation in U251-MG glioblastoma cells as detected by phospho-AKT western blots of RIPA cell extracts.* **A.** Western blots of RIPA cell extracts probed with indicated antibodies according to manufacture's specifications after 24 hours treatment with either DMSO control, 10µM Compound **14, B.** 30µM Compound **14,** or **C.** 50µM Compound **14**, as indicated. On the right is the quantitation of left side western anti-Phospho-Thr-308 (AKT P-Thr-308) and anti-phospho-Ser-473 (AKT P-Ser-473) bands is presented on the right, ns= not significant by unpaired t-test, n=3. These data are consistent with Compound 14 not altering AKT signaling in U251-MG cells, consistent with previous observations that PI3-kinase signaling in U251 cells can operate in an AKT-independent manner (24).

Supplemental Figure S15:

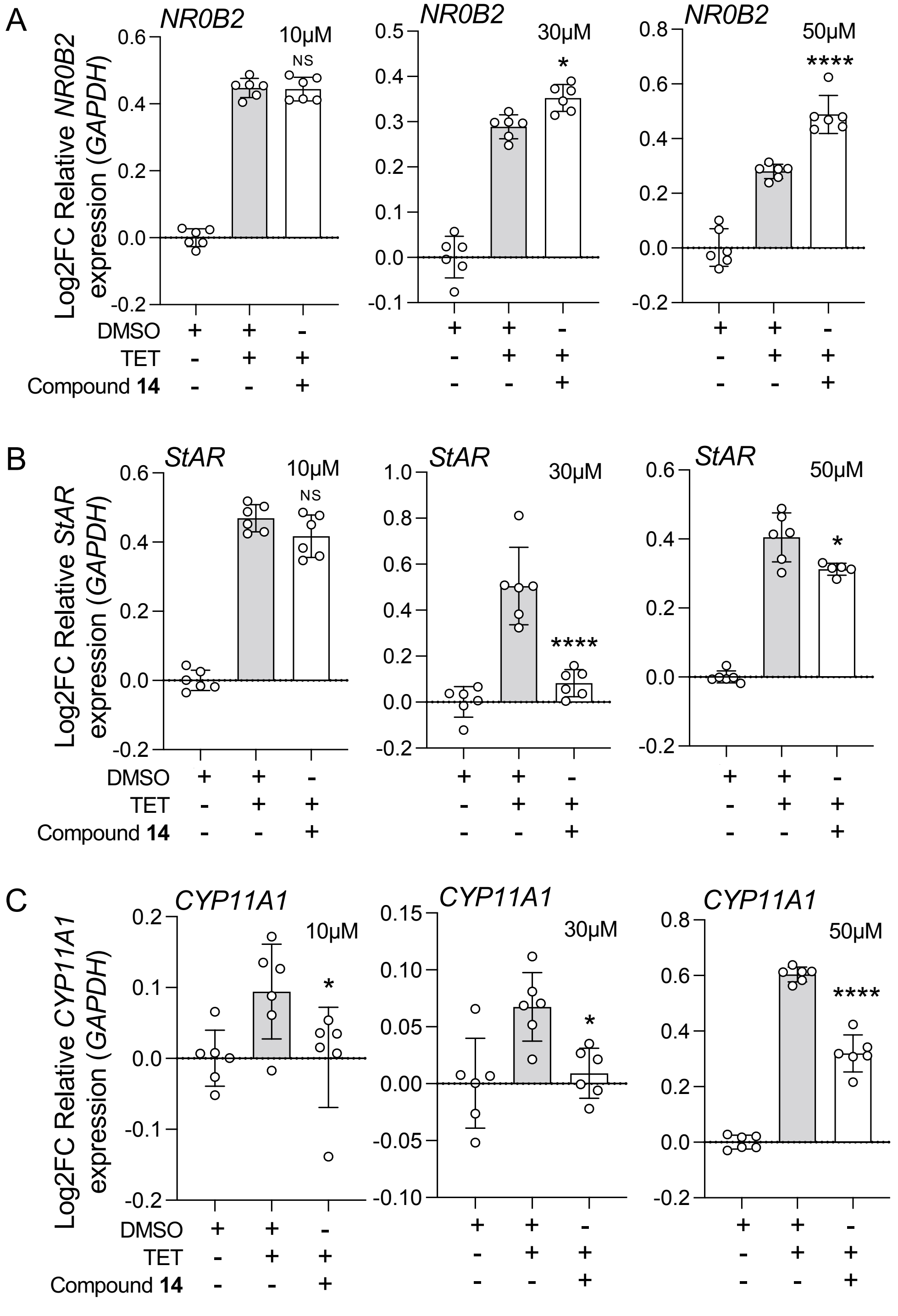

Figure S15. *Compound **14** regulates multiple SF-1 target genes in HEK293 cells.* RT-qPCR analysis of RNA from HEK293 cells bearing a tetracycline (Tet)-inducible SF-1. All treatments of Tet and Compound **14** were for 24 hours total before RNA extraction and qPCR performed for **A.** NR0B2, **B.** StAR and **C**. CYP11A1; ns = not significant, **p<0.05, ****p<0.0001* by unpaired t-test compared to DMSO + TET control (middle bar), for all panels n=3 biological replicates and 2 qPCR technical replicates, all data shown. These data suggest that Compounds 14 can regulate these target genes, which are well-established direct target genes of the nuclear receptor transcription factor SF-1.
